# A novel isoform of ATOH8 promotes the metastasis of breast cancer by regulating RhoC

**DOI:** 10.1093/jmcb/mjaa050

**Published:** 2020-10-13

**Authors:** Mengyao Xu, Shan Huang, Xiaoli Dong, Yanan Chen, Miao Li, Wen Shi, Guanwen Wang, Chongbiao Huang, Qiong Wang, Yanhua Liu, Peiqing Sun, Shuang Yang, Rong Xiang, Antao Chang

**Affiliations:** 1 School of Medicine, Nankai University, Tianjin 300071, China; 2 Department of Cancer Biology and Comprehensive Cancer Center, Wake Forest University Medical Center, Winston-Salem, NC 27157, USA; 3 International Collaborative Innovation Center of Medicine, Nankai University, Tianjin 300071, China; 4 Department of Pancreatic Cancer, Tianjin Medical University Cancer Institute and Hospital, National Clinical Research Center for Cancer, Key Laboratory of Cancer Prevention and Therapy, Tianjin 300060, China

**Keywords:** breast cancer, ATOH8, metastasis, RhoC

## Abstract

**Metastases are the main cause** of cancer-related mortality in breast cancer. Although significant progress has been made in the field of tumor metastasis, the exact molecular mechanisms involved in tumor metastasis are still unclear. Here, we report that ATOH8-V1, a novel isoform of ATOH8, is highly expressed in breast cancer and is a negative prognostic indicator of survival for patients. Forced expression of ATOH8-V1 dramatically enhances, while silencing of ATOH8-V1 decreases the metastasis of breast cancer cell lines. Moreover, ATOH8-V1 directly binds to the RhoC promoter and stimulates the expression of RhoC, which in turn enhances the metastasis of breast cancer. Altogether, our data demonstrate that ATOH8-V1 is a novel pro-metastatic factor that enhances cancer metastasis, suggesting that ATOH8-V1 is a potential therapeutic target for treatment of metastatic cancers.

## Introduction

Breast cancer is the most common type of cancer and the primary cause of cancer mortality in women worldwide (Bray et al., 2018; Siegel et al., 2019). Tumor metastasis is the major cause of death of cancer patients, responsible for >90% of all cancer deaths (Wan et al., 2013). Despite extensive studies focusing on tumor metastasis in the last decades, the exact molecular mechanisms for breast cancer invasion and metastasis are still unclear.

Basic helix–loop–helix (bHLH) transcription factors constitute a large eukaryotic protein family, called bHLH superfamily, which control numerous aspects of vertebrate organ development and functions (Massari and Murre, 2000; Ejarque et al., 2013). These factors are defined by the presence of a bHLH domain, in which the helix‒loop‒helix region mediates dimerization with a second bHLH protein to form a homo or heterodimer, and the positive charge enriched basic region mediates binding to DNA (Murre et al., 1994). *Atoh8* (*Hath6*) is a newly identified bHLH transcription factor, which is the sole mammalian member of the Net family within the atonal superfamily of bHLH factors. Unlike other members of the atonal superfamily that are encoded by a single exon, *Atoh8* displays an unique three-exon gene structure that is conserved from zebrafish to mammals (Inoue et al., 2001; Rawnsley et al., 2013). It has been reported that ATOH8 is an important regulator of the development of neurons, as well as the pancreas and kidney during embryonic development (Inoue et al., 2001; Ross et al., 2006; Lynn et al., 2008; Rawnsley et al., 2013; Fang et al., 2014).

Many of bHLH transcription factors, such as c-Myc (Kress et al., 2015; Stine et al., 2015), Twist (Kang and Massague, 2004; Cheng et al., 2008), HIF-1α (LaGory and Giaccia, 2016; Rankin and Giaccia, 2016), have been reported to play important roles in tumor initiation and progression (Bersten et al., 2013). However, the function of ATOH8 in tumor development is still unclear. A recently study reported that loss of ATOH8 increased the stemness of hepatocellular carcinoma (Song et al., 2015). Another study showed that down regulation of ATOH8 contributed to the malignant phenotype of nasopharyngeal carcinoma (Wang et al., 2016), suggesting a tumor suppression effect of ATOH8. However, the exact role and underlying molecular mechanism of ATOH8 in tumor metastasis is poorly understood. Here, we report that ATOH8-V1, a novel isoform of ATOH8 generated through a different splice pattern of the *Atoh8* gene, is highly expression in breast cancer, and is negative correlated with the survival of patients. Forced expression of ATOH8-V1 dramatically enhances the metastasis of breast cancer cell lines both *in vitro* and *in vivo*. Similarly, silence of ATOH8-V1 significantly decreased the metastasis of breast cancer cell lines. Moreover, ATOH8-V1 directly binds to the promoter of Ras homolog gene family, member C (RhoC), a pro-metastatic small G protein, and promotes its expression, thereby leading to enhanced breast cancer metastasis. These findings identify ATOH8-V1 as a novel pro-metastatic factor that mediates the metastasis of breast cancer.

## Results

### ATOH8-V1 is a novel isoform of ATOH8 in breast cancer cell lines

Recent studies have reported that the ATOH8 expression level is negatively correlated with stemness in hepatocellular carcinoma (Song et al., 2015), and that downregulation of ATOH8 contributes to the malignant phenotype of nasopharyngeal carcinoma (Wang et al., 2016), suggesting that ATOH8 is a tumor suppressor. However, our data revealed that ATOH8 was strongly expressed both in breast cancer cell lines and cancer tissues, and was positively correlated with the metastatic potential of these cells (Supplementary Figure S1A–C). Interestingly, results based on cBioPortal (Cerami et al., 2012; Gao et al., 2013) showed that amplification is the major alteration type of *atoh8* in multiple cancers (Supplementary Figure S1D). In breast cancer, most of cases with ATOH8 amplification are breast invasive ductal carcinoma (23 out of 24) with ER^−^/HER2^−^/PR^−^ (17 out of 24), an aggressive subgroup of breast cancer with poor prognosis (Supplementary Figure S1E). Moreover, data from the TCGA database indicated that patients with high ATOH8 expression had poorer long-term survival than these with low ATOH8 expression (Supplementary Figure S1F), suggesting an oncogenic activity of ATOH8, which is opposite to the results of previous studies. As *atoh8* is the only family member with three-exon gene structure (Inoue et al., 2001; Rawnsley et al., 2013), we reasoned that there may be different isoforms of ATOH8 in cells, and had opposite functions in tumor development.

**Figure 1:**
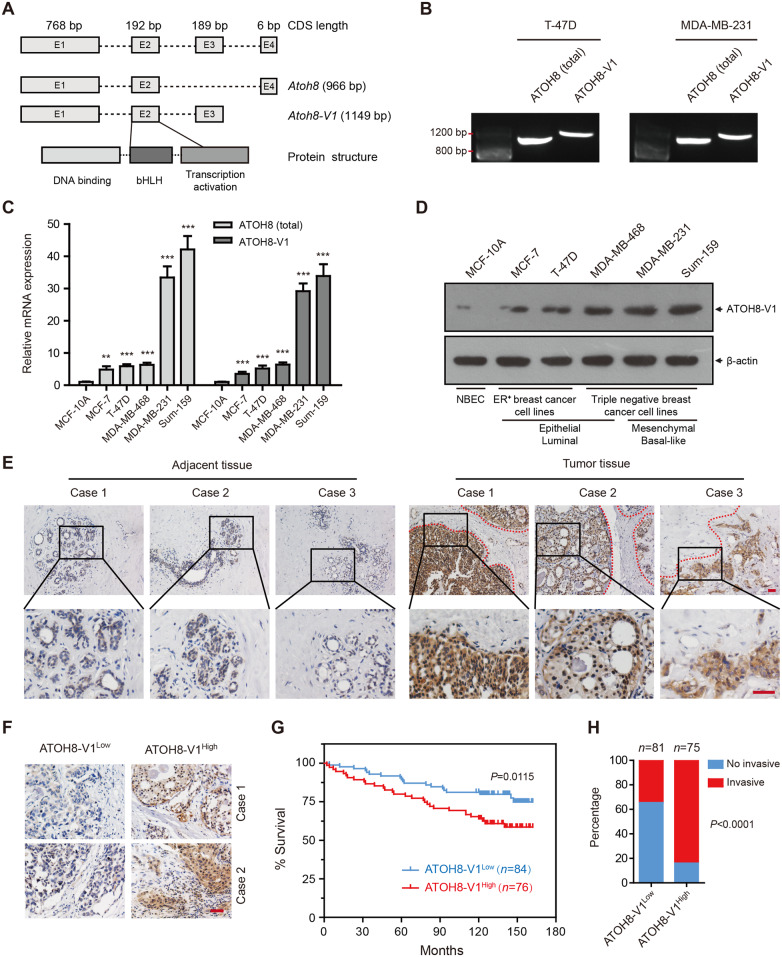
ATOH8-V1 is highly expressed in breast cancer and negatively correlated with the survival of patients. (**A**) Schematic diagram of the splice pattern of ATOH8-V1 and ATOH8. (**B**) RT-PCR analysis of the expression of ATOH8-V1 in breast cancer cell lines. (**C** and **D**) Real-time qPCR (**C**) and immunoblotting (**D**) analysis of ATOH8-V1 expression levels in breast cancer cell lines. **P <* *0.01 and ***P <* *0.001 for comparisons to MCF-10A groups using unpaired *t-*test. (**E**) IHC staining of ATOH8-V1 in cancer-adjacent and breast cancer tissues. (**F**) The criterion of high and low ATOH8-V1 expression levels in breast cancer tissues. (**G**) Survival analysis of patients in ATOH8-V1^low^ group and ATOH8-V1^high^ group. Kaplan‒Meier analysis is used for the estimation of overall survival. *P*-value is assessed by the log-rank test. (**H**) Statistical analysis of lymph node invasion between ATOH8-V1^low^ group and ATOH8-V1^high^ group. No invasive represents staging of N0 case, while invasive represents staging of N1‒N3 cases. *P-*value is calculated by Fisher’s exact test. CDS, coding sequence; E, exon; NBEC, nontumorigenic breast epithelial cell lines. Scale bar, 100 μm.

To test our hypothesis, we analyzed the genomic sequence of *atoh8* and found a potential splicing variant of the *atoh8* gene transcript, termed ATOH8-V1, which is longer and has an unique C-terminal domain as compared to the standard ATOH8 (Figure 1A). Then, we performed a reverse-transcription polymerase chain reaction (RT-PCR) assay to detect whether it is expressed in breast cancer cell lines. Interestingly, result indicated that ATOH8-V1 was indeed expressed both in T-47D and MDA-MB-231 cells (Figure 1B). Moreover, sequencing of the PCR products further confirmed ATOH8-V1 as a novel isoform of ATOH8 that was expressed in breast cancer cell lines.

### ATOH8-V1 is highly expressed in breast cancer cell lines and positively correlated with their metastatic potential

To study the role of ATOH8-V1 in breast cancer, we did a real-time quantitative PCR (qPCR) analysis to detect ATOH8-V1 expression in nontumorigenic breast epithelial (MCF-10A) and breast cancer cell lines, using a pair of specific primers that only target the unique C-terminal domain of ATOH8-V1. Interestingly, our data revealed that ATOH8-V1 was strongly expressed in breast cancer cell lines, and its expression level positively correlated with the metastatic potential of these cells (Figure 1C).

We next produced a specific antibody against ATOH8-V1 using a synthetic 14-amino acid (aa) peptide from the unique C-terminal domain as antigen, and then preformed an immunoblotting analysis to detect the expression of ATOH8-V1 in breast cancer cell lines. Notably, our data revealed that the expression level of ATOH8-V1 is higher in cell lines with strong metastatic potential than these with weak metastatic potential (Figure 1D), suggesting that ATOH8-V1 may play an important role in the metastasis of breast cancer cells.

### ATOH8-V1 is strongly expressed in human breast cancer tissues and is a negative prognostic indicator of survival for patients

To verify the applicability of the anti-ATOH8-V1 antibody in immunohistochemical (IHC) staining of human breast cancer tissues, we first performed a staining in cell culture blocks of MDA-MB-231 cells transduced with ATOH8-V1 shRNAs (Supplementary Figure S2A) and T-47D cells transduced with ATOH8-V1 or ATOH8 (Supplementary Figure S2B). Data indicated that the staining intensity of MDA-MB-231 cells with ATOH8-V1 knockdown were much weaker than the control cells (Supplementary Figure S2A). Moreover, as compared to control T-47D cells, ATOH8-V1-overexpressing cells, but not ATOH8-overexpressing cells, showed strong IHC staining (higher percentage of positive cells as well as staining intensity) (Supplementary Figure S2B). These results suggested that the ATOH8-V1 antibody could specifically detect ATOH8-V1 in IHC staining.

We then performed IHC staining of ATOH8-V1 in 10 cancer-adjacent and 160 breast cancer tissue samples to further investigate the function of ATOH8-V1 in breast cancer. Interestingly, we found that ATOH8-V1 was highly expressed in breast cancer tissues as compared to normal ductal epithelium of adjacent tissues (Figure 1E). Moreover, ATOH8-V1 was specifically expressed in tumor nest rather than the stroma (Figure 1E), suggesting that ATOH8-V1 may have some effect on the development and progression of breast cancer.

We next separated these tumor samples into high ATOH8-V1 expression group (ATOH8-V1^high^) and low ATOH8-V1 expression group (ATOH8-V1^low^) using the H-score (IHC score) system, which is calculated by percentage of positive-stained cells in each tumor tissues and their staining intensity (Figure 1F). Notably, survival analysis data demonstrated that patients in the ATOH8-V1^high^ group displayed a poorer outcome, with a lower survival rate as compared to the ATOH8-V1^low^ group. We recorded 74.85% of survival in the ATOH8-V1^low^ group at the end, as compared to only 58.61% of survival in the ATOH8-V1^high^ group (Figure 1G). Meanwhile, we also analyzed the association between ATOH8-V1 expression and clinicopathological features of breast cancer samples. Data indicated that the levels of ATOH8-V1 expression were not associated with Age, T staging, M staging, or TNM staging, but positively correlated with the Nodal staging (Table 1). Interestingly, patients in the ATOH8-V1^high^ group displayed higher grade of lymph node invasion as compared to the ATOH8-V1^low^ group (Figure 1H), suggesting a pro-metastatic effect of ATOH8-V1 in breast cancer.

**Table 1:** Association between ATOH8-V1 expression and clinicopathological features of breast cancer samples.

	Total	ATOH8-V1 expression		*P*-value
		Low	High	
Age (year)	160			0.263
<60		57	56	
≥60		27	20	
T staging	158			0.205
T1		23	13	
T2		55	52	
T2		5	10	
Nodal staging	156			<0.0001
N0		51	12	
N1		23	25	
N2		6	31	
N3		1	7	
M staging	160			N/A
M0		84	79	
TNM staging	160			0.3659
I		11	7	
I–II		16	11	
II		56	53	
II–III		1	4	
III		0	1	

To investigate whether ATOH8-V1 had the same effect in other types of cancer, we next detected the expression of ATOH8-V1 in a lung cancer tissue array with 273 cases, and an esophageal cancer tissue array with 131 cases. Similarly, ATOH8-V1 was highly expressed in tumor nest, and was a negative prognostic indicator of survival for patients (Supplementary Figure S2C–G).

Together, these results demonstrated that ATOH8-V1 was a negative prognostic indicator of survival for breast cancer patients, and may play a role in regulating the invasion of breast cancer cells.

### Forced expression of ATOH8-V1 enhances the metastasis of breast cancer cell lines

**Figure 2:**
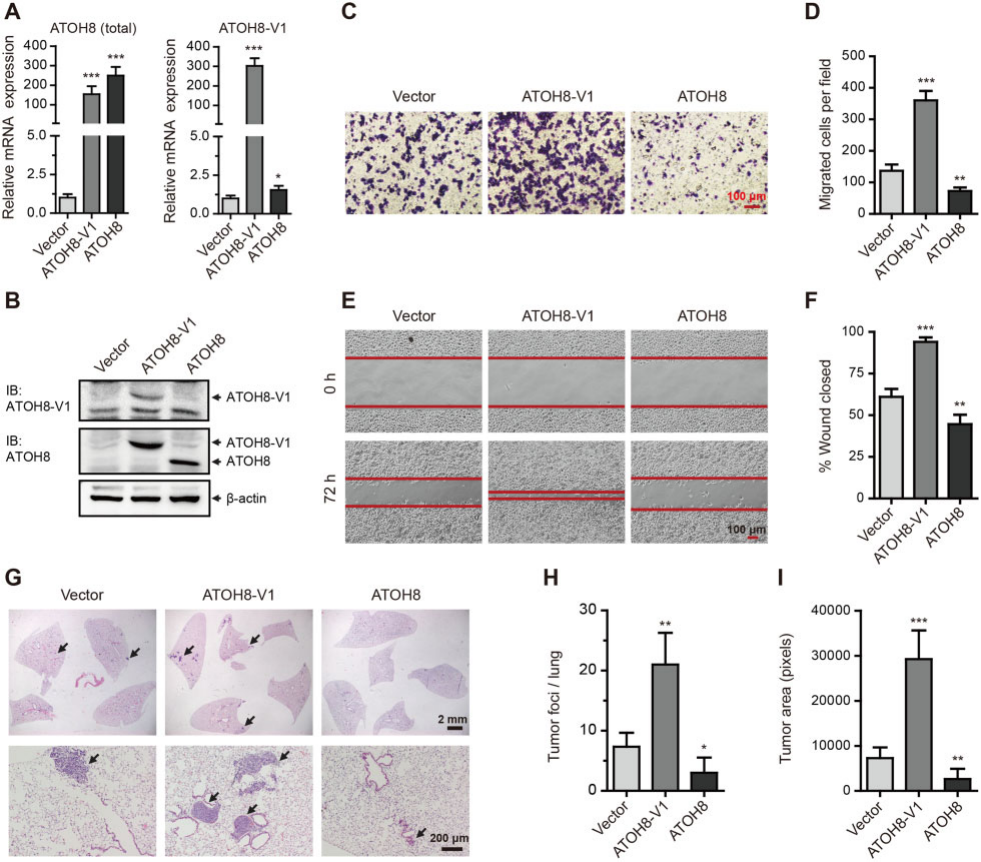
Forced expression of ATOH8-V1 promotes the metastasis of breast cancer cell lines. (**A** and **B**) qPCR (**A**) and immunoblotting (**B**) analysis of ATOH8-V1 and ATOH8 in T-47D cells transduced with ATOH8-V1 or ATOH8 overexpression. (**C** and **D**) Trans-well assay detecting the invasion of T-47D cells with ATOH8-V1 or ATOH8 overexpression. (**E** and **F**) Wound healing assay detecting the migration of T-47D cells with ATOH8-V1 or ATOH8 overexpression. (**G**) T-47D cells (5 × 10^6^) transduced with ATOH8-V1 or ATOH8 were subcutaneously injected into the second fat pad of female NOD-SCID mice. Orthotopic xenograft tumors were surgically removed when they were 500‒600 mm^3^ in size. After 12 weeks, mice were scarified and lung tissues were collected for H&E staining to detect lung metastasis. *n *=* *6. (**H**) Quantification of tumor foci numbers in lung tissues of mice. (**I**) Quantification of tumor area in lung tissues of mice. *P <* *0.05, **P <* *0.01, and ***P <* *0.001 for comparisons with Vector groups using unpaired *t-*test.

As ATOH8-V1 has potential to regulate metastasis, we next ectopically expressed ATOH8-V1 in low metastatic T-47D and MDA-MB-468 cells by lentiviral expression system (Figure 2A and B; Supplementary Figure S3A and B), and then performed trans-well assay and wound healing assay to detect the invasion and migration ability of these cells. Notably, results indicated that forced expression of ATOH8-V1 dramatically enhanced the invasion (Figure 2C and D; Supplementary Figure S3C and D) and migration (Figure 2E and F; Supplementary Figure S3E and F) of T-47D and MDA-MB-468 cells, while ATOH8 displayed an opposite effect, with a little inhibition. Interestingly, neither ATOH8 nor ATOH8-V1 obviously affected the proliferation (Supplementary Figure S4A and B) and apoptosis (Supplementary Figure S4C and D) of breast cancer cell lines, suggesting that ATOH8-V1 specifically regulates the process of tumor cell metastasis.

To confirm these *in vitro* results, 6-week-old female NOD-SCID mice were subcutaneously injected in the second fat pad with 5 × 10^6^ of T-47D or MDA-MB-468 cells transduced with ATOH8-V1, ATOH8, or vector control. Twelve weeks after injection, mice were scarified, and lung tissues were collected and analyzed by hematoxylin and eosin (H&E) staining to detect lung metastasis. Interestingly, there was no obvious difference in primary tumor growth among all the three groups, suggesting that neither ATOH8 nor ATOH8-V1 had any effect on growth of xenograft tumors in mice (Supplementary Figure S4E and F). However, as compared to control mice (vector control), mice seeded with ATOH8-V1-overexpressing breast cancer cell lines had more tumor foci and tumor area in the lung tissue, while mice seeded with ATOH8-overexpressing breast cancer cell lines showed an opposite phenotype, with less tumor foci and tumor area (Figure 2G–I; Supplementary Figure S3G–I), suggesting an antagonistic action between these two isoforms. These results demonstrate that forced expression of ATOH8-V1 significantly promotes the metastasis of breast cancer cell lines.

### Silencing of ATOH8-V1 inhibits the metastasis of breast cancer cell lines

**Figure 3:**
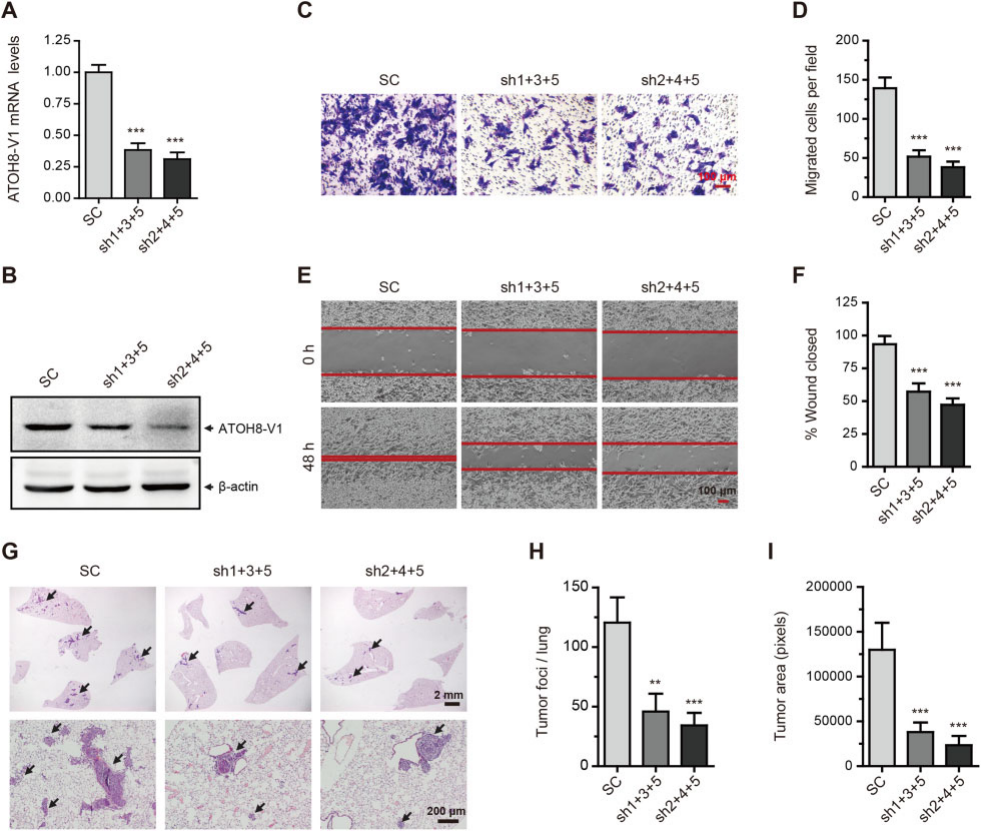
Silencing of ATOH8-V1 inhibits the metastasis of breast cancer cell lines. (**A** and **B**) qPCR (**A**) and immunoblotting (**B**) analysis of the knockdown efficiency of ATOH8-V1 in MDA-MB-231 cells transduced with ATOH8-V1 shRNAs. (**C** and **D**) Trans-well assay detecting invasion of MDA-MB-231 cells with ATOH8-V1 knockdown. (**E** and **F**) Wound healing assay detecting migration of MDA-MB-231 cells with ATOH8-V1 knockdown. (**G**) MDA-MB-231 cells (2 × 10^6^) transduced with ATOH8-V1 shRNAs were subcutaneously injected into the second fat pad of female NOD-SCID mice. Orthotopic xenograft tumors were surgically removed when they were 500‒600 mm^3^ in size. After 9 weeks, mice were scarified and lung tissues were collected for H&E staining to detect lung metastasis. *n *=* *6. (**H**) Quantification of tumor foci numbers in lung tissues of mice. (**I**) Quantification of tumor area in lung tissues of mice. **P <* *0.01 and ***P <* *0.001 for comparisons with shRNA control (SC) groups using unpaired *t-*test.

To further investigate the pro-metastatic effect of ATOH8-V1 in breast cancer, we next silenced ATOH8-V1 in MDA-MB-231 and Sum-159 cells with high metastatic potential by lentivirus-encoded shRNAs that specifically target the unique C-terminal domain of ATOH8-V1 (Figure 3A and B; Supplementary Figure S5A and B). Notably, silencing of ATOH8-V1 significantly inhibited the invasion (Figure 3C and D; Supplementary Figure S5C and D) and migration (Figure 3E and F; Supplementary Figure S5E and F) ability of MDA-MB-231 and Sum-159 cells, which was consistent with the overexpression results.

We then detected lung metastasis of MDA-MB-231 and Sum-159 cells transduced with ATOH8-V1 shRNAs. MDA-MB-231 (2 × 10^6^) or Sum-159 (1 × 10^6^) cells were subcutaneously injected in the second fat pad of NOD-SCID mice. Nine weeks after injection, mice were scarified, and lung tissues were collected and analyzed by H&E staining to detect lung metastasis. Consistently, silencing of ATOH8-V1 dramatically reduced the number and area of tumor foci in lung tissues of mice (Figure 3G–I; Supplementary Figure S5G–I). Altogether, these results demonstrate that ATOH8-V1 displays a strong pro-metastatic effect in breast cancer cell lines.

### ATOH8-V1 directly regulates the expression of RhoC in breast cancer cell lines

Previous studies have identified ATOH8 as a bHLH transcription factor that may regulate the expression of genes in the process of organism development (Inoue et al., 2001; Ross et al., 2006; Lynn et al., 2008; Rawnsley et al., 2013). To investigate the molecular mechanism of ATOH8-V1 involved in tumor metastasis, we performed a chromatin immunoprecipitation (ChIP)−Seq assay in MDA-MB-231 cells using the anti-ATOH8-V1 antibody to search for potential downstream effectors of ATOH8-V1. Interestingly, the ChIP−Seq data revealed that 30.73% (63 out of 205) of candidates (peak classification as promoter, *P*-value <1 × 10^−5^ and fold-enrichment >3) were tumor-associated genes, and most of them displayed tumor promotion action, suggesting that ATOH8-V1 played an important role on tumor progression.

We next chose Sox5, RhoC, PLA2G7, PDE7A, and HoxB13 as the potential downstream effectors of ATOH8-V1 for further verification, based on their fold-enrichment in the ChIP−Seq analysis (Supplementary Figure S6A and B) and their pro-metastatic effect in tumor as indicated by previous studies (Clark et al., 2000; Vainio et al., 2011; Shah et al., 2013; Renjie and Haiqian, 2015; Yamamoto et al., 2015). To investigate whether these five genes could be transcriptionally regulated by ATOH8-V1, we performed qPCR assay to detect the expression of these genes in T-47D cells transduced with ATOH8-V1 or ATOH8. Notably, the transcription levels of these five genes were promoted by ATOH8-V1 whereas inhibited by ATOH8 (Supplementary Figure S6C). Among them, Sox5 and RhoC had the highest induction by ATOH8-V1.

Sox5 has been reported to enhance tumor metastasis by promoting epithelial–mesenchymal transition (EMT) (Renjie and Haiqian, 2015; Wang et al., 2015). However, our data revealed that ATOH8-V1 did not obviously affect the EMT phenotypes in breast cancer cell lines on both morphological (Supplementary Figure S6D and E) and molecular (Supplementary Figure S6F and G) levels. We thus focused our attentions on RhoC.

**Figure 4:**
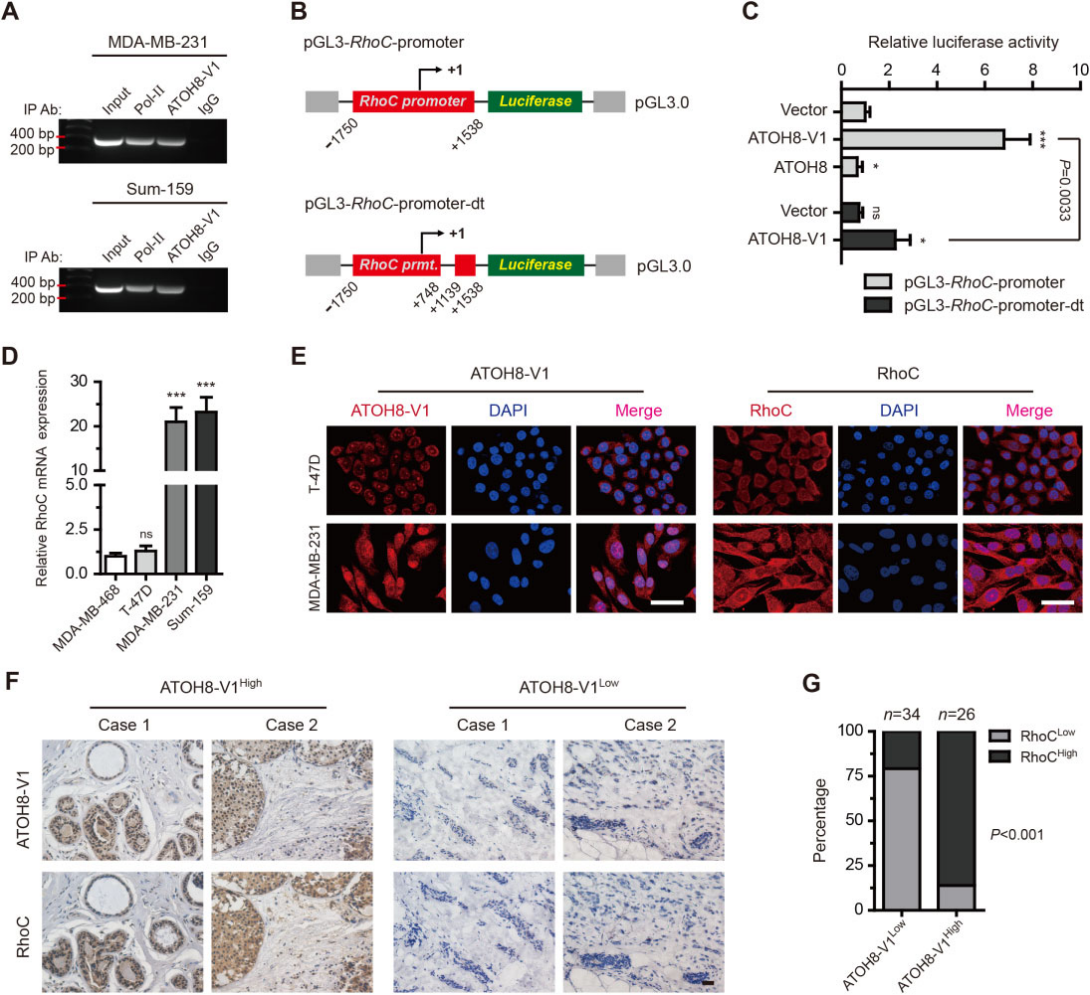
RhoC is the major downstream effector of ATOH8-V1 in breast cancer metastasis. (**A**) ChIP analysis of the binding of ATOH8-V1 to the RhoC promoter in breast cancer cell lines using the anti-ATOH8-V1 antibody. Anti-Pol II antibody was used as positive control, while normal IgG was used as negative control. (**B** and **C**) Dual-luciferase analysis of the activity of RhoC promoters in HEK-293T cells transfected with ATOH8-V1, ATOH8, or control vector. ns, not significant; *P *>* *0.05, *P <* *0.05, and ***P <* *0.001 for comparisons with Vector groups using unpaired *t-*test. (**D**) qPCR analysis of the mRNA levels of RhoC in breast cancer cell lines. ns, not significant (*P *>* *0.05); ***P <* *0.001 for comparisons with the first lane using unpaired *t-*test. (**E**) Immunofluorescent staining detecting the expression of ATOH8-V1 and RhoC in T-47D and MDA-MB-231 cells. (**F**) IHC staining of ATOH8-V1 and RhoC in serial sections of breast cancer tissues. (**G**) Correlation analysis of ATOH8-V1 and RhoC expression in breast cancer tissues. *P-*value is calculated by Fisher’s exact test.

RhoC is a small signaling G protein of the Rho family, which has been reported to play a critical role in tumor mobility, invasion, and metastasis (Clark et al., 2000; Lang et al., 2017; Guan et al., 2018). Our ChIP−Seq data showed that ATOH8-V1 can specifically bind to a region near the transcription start site of the RhoC promoter (Supplementary FigureS7A and B). To confirm this result, we performed a ChIP assay in MDA-MB-231 and Sum-159 cells using the anti-ATOH8-V1 antibody. Notably, we found that that fragment of the RhoC promoter was effectively coimmunoprecipitated with ATOH8-V1 both in MDA-MB-231 and Sum-159 cells (Figure 4A), suggesting that ATOH8-V1 can directly bind to this region of the RhoC promoter.

To study whether ATOH8-V1 enhances the transcription activity of the RhoC promoter, we cloned a 3-kb fragment of the RhoC promoter into pGL3.0 vector, and performed a dual-luciferase assay to detect activity of this promoter (Figure 4B). Notably, ATOH8-V1 dramatically increased the transcription activity of the RhoC promoter, while ATOH8 displayed an inhibitory effect (Figure 4C). Interestingly, deletion of the binding region of ATOH8-V1 on the RhoC promoter abolished ATOH8-V1-induced promotion of transcription activity (Figure 4C), suggesting that ATOH8-V1 enhances the transcription of RhoC by binding to this region of the promoter.

To further investigate the relationship between ATOH8-V1 and RhoC, we detected their expression in breast cancer cell lines at both mRNA and protein levels. qPCR data indicated that the expression pattern of RhoC mRNA was similar to ATOH8-V1 in breast cancer cell lines (Figures 1C and 4D). Moreover, forced expression of ATOH8-V1 dramatically increased the mRNA and protein levels of RhoC in T-47D and MDA-MB-468 cells, while ATOH8 overexpression displayed an inhibitory effect. Whereas silencing of ATOH8-V1 heavy reduced the expression of RhoC in MDA-MB-231 and Sum-159 cells (Supplementary Figure S7C and D). Interestingly, Immunofluorescence staining showed that ATOH8-V1 was highly expressed in MDA-MB-231 cells with mainly nucleus location, while less expressed in T-47D cells with cytoplasm localization (Figure 4E).

To verify these results, we next performed an IHC staining of ATOH8-V1 and RhoC in serial sections of breast cancer tissues. Data indicated that tumor tissues with ATOH8-V1 high expression also displayed a strong staining of RhoC (Figure 4F and G), suggesting a positive correlation of expression between ATOH8-V1 and RhoC. Together, these results demonstrate that ATOH8-V1 directly binds to the promoter of RhoC and positively regulates its expression.

### Silencing of RhoC abolishes the pro-metastatic effect of ATOH8-V1 in breast cancer cell lines

**Figure 5:**
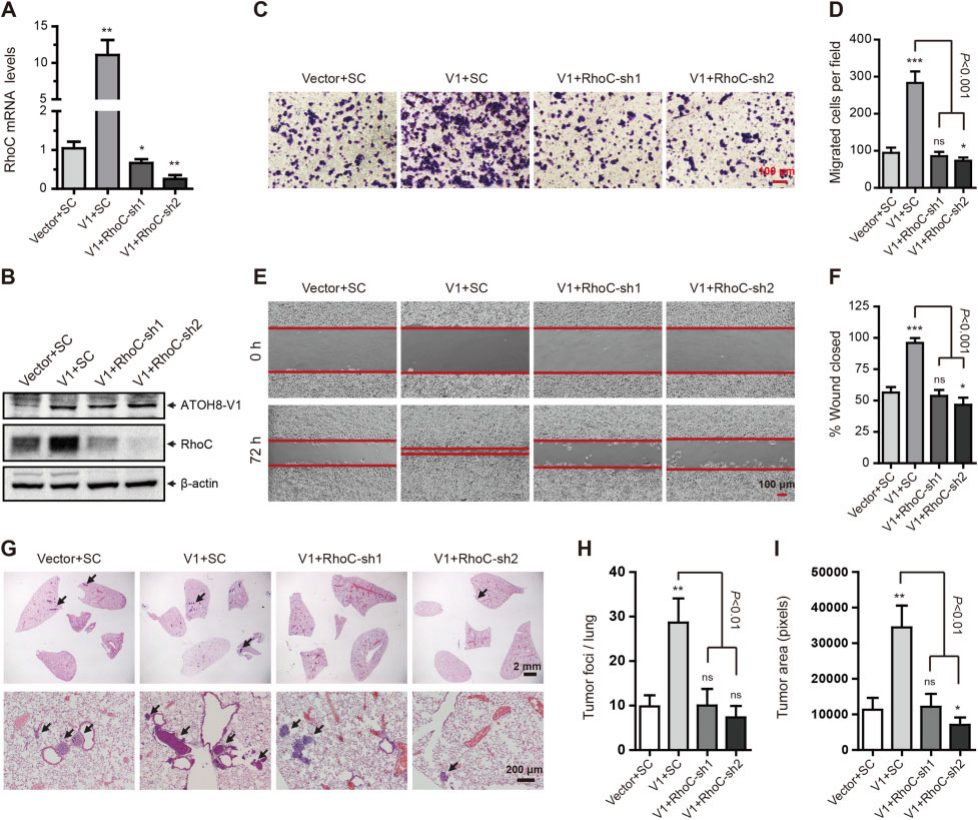
Silencing of RhoC abolishes the pro-metastatic effect of ATOH8-V1 in breast cancer cell lines. (**A**) qPCR analysis of RhoC mRNA levels in ATOH8-V1-overexpressing T-47D cells transduced with RhoC shRNA. (**B**) Immunoblotting analysis of ATOH8-V1 and RhoC in ATOH8-V1-overexpressing T-47D cells transduced with RhoC shRNA. (**C** and **D**) Trans-well assay detecting the invasion of ATOH8-V1-overexpressing T-47D cells transduced with RhoC shRNA. (**E** and **F**) Wound healing assay detecting migration of ATOH8-V1-overexpressing T-47D cells transduced with RhoC shRNAs. (**G**) H&E staining of lung tissue slices detecting lung metastasis of ATOH8-V1-overexpressing T-47D cells transduced with RhoC shRNAs in mice. *n *=* *6. (**H**) Quantification of tumor foci numbers in lung tissues of mice. (**I**) Quantification of tumor area in lung tissues of mice. ns, not significant (*P *>* *0.05); *P <* *0.05, **P <* *0.01, and ***P <* *0.001 for comparisons with Vector+SC groups using unpaired *t-*test.

To investigate whether RhoC is the major downstream effector of ATOH8-V1 in the metastasis of breast cancer, we silenced RhoC in T-47D and MDA-MB-468 cells with ATOH8-V1 overexpression (Figure 5A and B; Supplementary Figure S8A and B), and then detected the migration and invasion ability of these cell lines using wound healing and Trans-well assay, respectively. Interestingly, our data indicated that silencing of RhoC abolished ATOH8-V1-induced promotion of invasion (Figure 5C and D; Supplementary Figure S8C and D) and migration (Figure 5E and F; Supplementary Figure S8E and F) both in T-47D and MDA-MB-468 cells, suggesting that RhoC is the major downstream effector of ATOH8-V1 in tumor migration and invasion.

To further verify these results *in vivo*, we constructed lung metastasis models of these cells in female NOD-SCID mice and performed H&E staining to detect metastasis foci in lung tissues at 12 weeks after tumor cell injection. Consistently, forced expression of ATOH8-V1 dramatically enhanced lung metastasis of T-47D and MDA-MB-468 cells in mice, while silencing of RhoC in these cells abolished the promotion of lung metastasis by ATOH8-V1, as indicated by less tumor foci and tumor area in the lung of mice injected with ATOH8-V1-overexpressing breast cancer cell lines transduced with RhoC shRNA (Figure 5G–I; Supplementary Figure S8G–I), suggesting that RhoC is the major downstream effector of ATOH8-V1 in tumor metastasis.

### Forced expression of RhoC restores the metastatic ability of breast cancer cell lines with ATOH8-V1 knockdown

**Figure 6:**
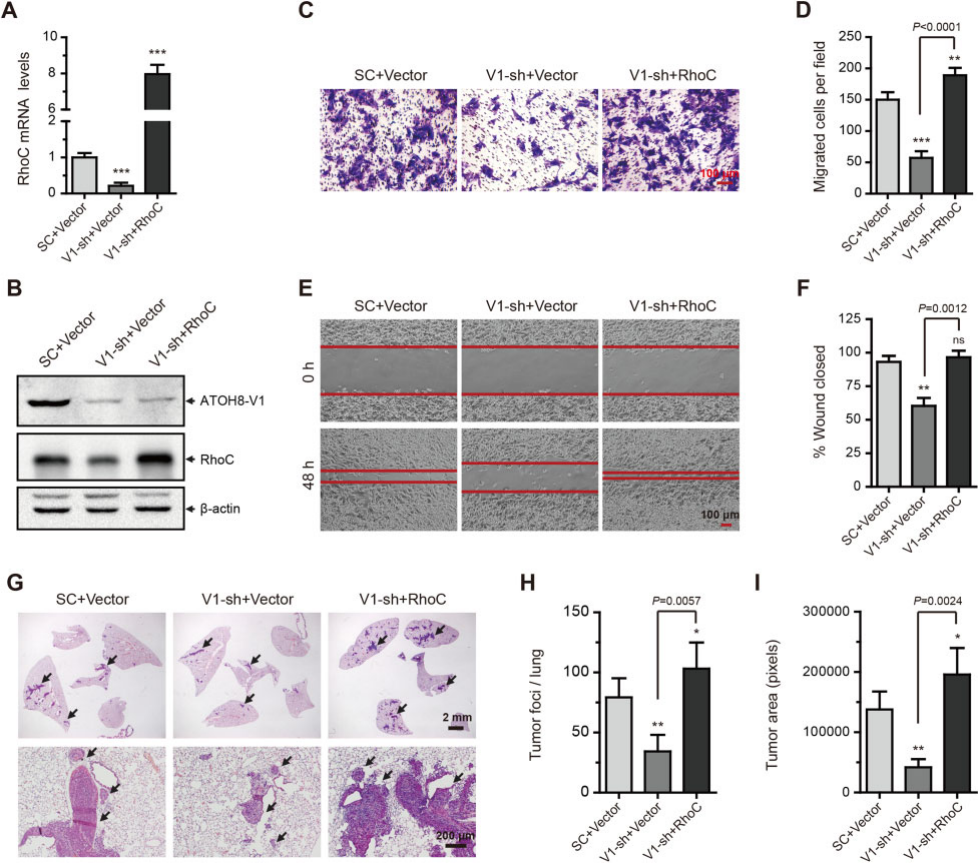
Forced expression of RhoC restores the metastatic ability of ATOH8-V1-silenced breast cancer cell lines. (**A**) qPCR analysis of RhoC mRNA levels in ATOH8-V1-silenced MDA-MB-231 cells transduced with RhoC. (**B**) Immunoblotting analysis of ATOH8-V1 and RhoC in ATOH8-V1-silenced MDA-MB-231 cells transduced with RhoC. (**C** and **D**) Trans-well assay detecting invasion of ATOH8-V1-silenced MDA-MB-231 cells transduced with RhoC. (**E** and **F**), Wound healing assay detecting migration of ATOH8-V1-silenced MDA-MB-231 cells transduced with RhoC. (**G**) H&E staining of lung tissue slices detecting lung metastasis of ATOH8-V1-silenced MDA-MB-231 cells transduced with RhoC in mice. *n *=* *6. (**H**) Quantification of tumor foci numbers in lung tissues of mice. (**I**) Quantification of tumor area in lung tissues of mice. ns, not significant (*P *>* *0.05); *P <* *0.05, **P <* *0.01, and ***P <* *0.001 for comparisons with SC+Vector groups using unpaired *t-*test.

To further investigate the regulatory relationships between ATOH8-V1 and RhoC in tumor metastasis, we next ectopically expressed RhoC in MDA-MB-231 and Sum-159 cells with ATOH8-V1 knockdown (Figure 6A and B; Supplementary Figure 9A and B). Consistently, trans-well and wound healing assay indicated that forced expression of RhoC restored invasion (Figure 6C and D; Supplementary Figure S9C and D) and migration (Figure 6E and F; Supplementary Figure S9E and F) that had been reduced due to ATOH8-V1 knockdown in breast cancer cell lines.

We next detected the lung metastasis formation of these cell lines in NOD-SCID mice. Consistently, silencing of ATOH8-V1 significantly inhibited lung metastasis of MDA-MB-231 and Sum-159 cells in mice, while forced expression of RhoC in these cells restored their metastatic ability that had been reduced due to ATOH8-V1 knockdown (Figure 6G–I; Supplementary Figure S9G–I). Collectively, these results demonstrate that ATOH8-V1 enhances the metastasis of breast cancer cell lines by directly promoting the expression of RhoC.

## Discussion

Breast cancer is the most common cancer and the leading cause of cancer death among women worldwide. Metastasis is the major cause of cancer-related patients’ death despite that significant progress has been made in this field. In this study, we have defined a role of ATOH8-V1, a novel splicing variant of *atoh8* gene, in promoting the metastasis of breast cancer. Moreover, this pro-metastatic effect of ATOH8-V1 is mainly mediated by RhoC, a small signaling G protein that is required for tumor metastasis (Clark et al., 2000; Lang et al., 2017).

ATOH8 is a typical bHLH transcription factor that expresses in a variety of organs and tissues during embryonic development, and is involved in cell differentiation of these tissues (Rawnsley et al., 2013). Previous studies reported that ATOH8 seemed to be a tumor suppresser in hepatocellular carcinoma (Song et al., 2015) and nasopharyngeal carcinoma (Wang et al., 2016). In our study, we have identified ATOH8-V1 as a novel isoform of ATOH8 generated through a different splice pattern. Moreover, our data indicates that ATOH8-V1 is strongly expressed in breast cancer and displays a strong pro-metastatic activity. However, the underlying mechanism involved in regulation of this alternative splicing process that generates ATOH8-V1 and that specifically enhances the expression of ATOH8-V1 in breast cancer is still unknown.

ATOH8-V1 is longer than the standard ATOH8, with an unique 60aa C-terminal domain. According to the protein structure, the C-terminal domain of ATOH8 is the transcription activation domain, which mediates interaction with transcriptional coactivators to form transcriptional complex, and then initiates transcription of target genes. A previous report showed that ATOH8 only had a weak transcriptional activity and might even serve as an negative regulator of transcription (Ejarque et al., 2013). Interestingly, our data reveal that ATOH8-V1 enhances the expression of downstream genes, and may display a strong transcriptional activity. We reason that the unique C-terminal domain of ATOH8-V1 may be responsible for efficient binding to certain transcriptional coactivators, thus leading to initiation of transcription of downstream genes, while ATOH8, which lacks this domain, cannot mediate efficient transcription because it does not interact with the transcriptional coactivators that specifically interact with ATOH8-V1. However, the identity of the transcriptional coactivators that specifically interact with ATOH8-V1 is currently unclear and needs to be defined by further studies.

Moreover, although previous studies showed that ATOH8 displays a weak transcription activity, our data reveal that forced expression of ATOH8 decreases the expression of downstream genes and inhibits breast cancer metastasis. As a bHLH transcription factor, ATOH8 can form homo- or hetero-dimer with other bHLH factor to regulate the transcription of target genes. It is likely that exogenously expressed ATOH8 may form heterodimer with endogenous ATOH8-V1 to prevent it from binding to the transcriptional coactivators, thus leading to inhibition of transcription of downstream target genes and suppression of tumor metastasis. This notion is currently under investigation.

RhoC is a small signaling G protein, and is a member of the Rac subfamily of the Rho family of GTPases (Ridley, 2006; Lang et al., 2017). Previous studies have reported that overexpression of RhoC enhances cell motility, rendering cells to become invasive (Ikoma et al., 2004). Moreover, although RhoC-deficient mice can still develop tumors, these tumors fail to metastasize, arguing that RhoC is required for tumor metastasis (Hakem et al., 2005). Interestingly, our data indicated that ATOH8-V1 directly binds to the RhoC promoter and stimulates its expression, thereby enhancing the metastasis of breast cancer. Furthermore, deletion of the ATOH8-V1-binding region from the RhoC promoter prevents ATOH8-V1 from stimulating transcription of RhoC. bHLH transcription factors typically bind to a consensus sequence called E-box (CANNTG), with the exception that some bHLH proteins bind to other sequences that are similar to the E-box (Skinner et al., 2010; Bersten et al., 2013). Although we have identified several potential-binding motifs of ATOH8-V1 through ChIP−Seq (Supplementary Figure S7A), the exact-binding motif of ATOH8-V1 in the RhoC promoter is still unclear and needs to be further defined.

In conclusion, our findings identify ATOH8-V1, a novel isoform of ATOH8, as a pro-metastatic factor that enhances the metastasis of breast cancer. Thus, this study provides novel insights into the exact role of the *atoh8* gene in tumor development, and helps to better understand the RhoC signaling in tumor metastasis.

## Materials and methods

### Tissue samples

Breast cancer tissue array was purchase from Shanghai Outdo Biotech Co., Ltd. Lung cancer and esophageal cancer tissue samples were obtained from Tianjin Tumor Hospital. This study was approved by the institutional ethics committees at Tianjin Tumor Hospital and Medical school of Nankai University.

### Animal studies

Six-week-old female NOD-SCID mice were purchased from Beijing HFK Bioscience Co., Ltd and maintained under the guidelines on laboratory animals of Nankai University.

For detecting lung metastasis, NOD-SCID mice were subcutaneously injected with Sum-159 (1 × 10^6^), MDA-MB-231 (2 × 10^6^), T-47D (5 × 10^6^), and MDA-MB-468 (5 × 10^6^) cells, respectively, in the second fat pad. Tumor volumes were measured every 3‒4 days. Tumor volume was calculated by Length×Width^2^/2. Orthotopic xenograft tumors were surgically removed when they were 500‒600 mm^3^ in size. At 9‒12 weeks after injection, mice were scarified and lung tissues were collected, fixed in 4% paraformaldehyde, paraffin-embedded, and sectioned in 7-μm thickness for histopathological analysis.

### Antibodies

The antibodies used for western blot analysis, immunostaining assays, and ChIP assays are provided in Supplementary material.

### Cell culture

All the human breast cancer cell lines were purchased from ATCC. T-47D cells and Sum-159 cells were cultured in Dulbecco's Modified Eagle's Medium containing 10% fetal bovine serum (FBS), 1% sodium pyruvate, and 1% penicillin–streptomycin (P/S), at 37°C in a humidified incubator containing 5% CO_2_. MDA-MB-231 cells and MDA-MB-468 were cultured in L-15 Medium containing 10% FBS and 1% P/S, at 37°C in a humidified incubator containing 0% CO_2_.

### RNA analysis

Total RNA was transcribed to cDNA with the First-Strand cDNA Synthesis system (TransGen Biotech). SybrGreen-based real-time qPCR was performed using the Thermal Cycler (Bio-Rad) following the manufacturer’s instructions. qPCR primers are listed in Supplementary Table S1.

### Vector construction and virus production

All the genes were amplified by RT-PCR and then inserted into the pLV-cDNA cloning vector (Biosettia) with indicated restriction enzyme. Single-stranded shRNA was annealed and then ligated to the pLV-RNAi vector (Biosettia). Recombinant lentiviruses were packaged and transduced into cells as described before (Chang et al., 2015).

### Western blot analysis

Cells were collected and lysed in RIPA buffer for 30 min on ice and then centrifuged at full speed for 10 min at 4°C. The supernatants were collected and subjected to SDS‒PAGE followed by immunoblotting as described before (Chang et al., 2015).

### Cell migration and invasion assays

For wound healing assay, breast cancer cell lines were cultured in a 6-well culture plate for 24 h until 90%‒95% confluent. Wound line was created by scratching the plate with a 10-μl micropipette tip. Then, cells were washed with phosphate-buffered saline (PBS) to remove the floating cells and cultured in basic medium containing 1% FBS for the indicated time. The average width of the gaps was calculated from the image taken with a microscope.

For trans-well assay, modified 24-well (8.0-μm pore size) chamber with matrix gel (Millipore) was inserted into a 24-well plate containing 500 μl complete medium. Then, 1 × 10^5^ cells in 200 μl basic medium containing 1% FBS were seeded into the top chamber and cultured for the indicated time. Later, the inner of the chamber was carefully scraped by a swab, and stained with crystal violet for 2 h. The number of invasive cells was counted from the image taken with a microscope.

### Preparation of cell culture blocks as tissue microarray paraffin block donors

When 50%‒75% confluent, cells were collected and fixed in 10% (*v*/*v*) phosphate-buffered formalin at room temperature for overnight. Fixed cells were collected by centrifugation at 500 *g* for 10 min and washed once with PBS. Cell pellets were resuspended with an equal volume of 0.8% agarose (prepared in PBS) at 42°C and then transferred into a microfuge tube that was previously filled with agarose. After the agarose solidified, cell plugs were carefully removed from the microfuge tube, sectioned in half to generate two cell blocks, and then processed into paraffin blocks using standard tissue processing.

### Histological analysis

IHC analysis of paraffin-embedded sections was performed using the Envision Kit (Dako) following the manufacturer’s protocols. Sections were analyzed by microscopy.

IHC staining was evaluated blindly by two independent investigators who were unaware of the patients’ clinical information. The intensity of stained cells was scored as 0, 1, 2, or 3, representing negative, weak, moderate, or strong, respectively. Positive-stained cells were counted for percentage, and a final H-score was calculated as follow: (% weak-stained cells × 1+ % moderate-stained cells × 2+ % strong-stained cells × 3)×100. H-scores ranged from 0 to 300 (Chen et al., 2015). For ATOH8-V1 staining, an H-score ≥150 points was defined as high expression, whereas an H-score < 150 points was defined as low expression.

To detect lung metastasis, mouse lung tissue sections were stained with H&E and then analyzed by microscopy.

### Immunofluorescence

Breast cancer cell lines were grown on sterilized glass-bottom microwell dishes (MatTek Corporation) for 2 days and then fixed and permeated. Staining was performed as described in Supplementary material.

### ChIP and ChIP−Seq assays

Cells were seeded in a 150-mm dish, cultured for 2 days to reach ∼80% confluent, and then directly cross-linked with 1% formaldehyde for 15 min at room temperature. The cells were then washed with PBS, collected with scraper, and subjected to ChIP assay using the MAGnify^TM^ Chromatin Immunoprecipitation System (Invitrogen) with the antibody specifically against ATOH8-V1 (10 μg per reaction).

After purification, DNA samples were sequenced using the Illumina Sequencing Platform. Massive parallel DNA sequencing and data analysis were performed by KangChen Bio-tech, Inc.

To verify the binding of ATOH8-V1 to the RhoC promoter, DNA samples were amplified by PCR using a pair of primers derived from the binding region of ATOH8-V1 on the RhoC promoter. Sequences of primers are listed in Supplementary Table S1.

### Dual-luciferase assay

Cells were co-transfected with the wild-type or deletion mutants of human RhoC promoters and ATOH8-V1 or ATOH8 expression plasmid or empty vector as control in 24-well plates. Lysates were prepared at 24 h after transfection, and luciferase activities were measured using the Dual-luciferase Reporter Assay System (Promega) according to the manufacturer’s protocols. Firefly luciferase activities were normalized to the values for Renilla luciferase.

### Statistical analysis

Statistical significance was determined by *t*-test, Kaplan‒Meier analysis, or Fisher’s exact test using the GraphPad Prism 5.01. Data were expressed as means ±standard deviation. *P*-values <0.05 were considered significant.

## Supplementary material

Supplementary material is available at *Journal of Molecular Cell Biology* online.

## Supplementary Material

mjaa050_Supplementary_Data

## References

[mjaa050-B1] Bersten D.C. , Sullivan A.E., Peet D.J., et al (2013). bHLH–PAS proteins in cancer. Nat. Rev. Cancer 13, 827–841. 24263188 10.1038/nrc3621

[mjaa050-B2] Bray F. , Ferlay J., Soerjomataram I., et al (2018). Global cancer statistics 2018: GLOBOCAN estimates of incidence and mortality worldwide for 36 cancers in 185 countries. CA Cancer J. Clin. 68, 394–424. 30207593 10.3322/caac.21492

[mjaa050-B3] Cerami E. , Gao J., Dogrusoz U., et al (2012). The cBio cancer genomics portal: an open platform for exploring multidimensional cancer genomics data. Cancer Discov. 2, 401–404. 22588877 10.1158/2159-8290.CD-12-0095 PMC3956037

[mjaa050-B4] Chang A. , Chen Y., Shen W., et al (2015). Ifit1 protects against lipopolysaccharide and D-galactosamine-induced fatal hepatitis by inhibiting activation of the JNK pathway. J. Infect. Dis. 212, 1509–1520. 26459629 10.1093/infdis/jiv221 PMC6373839

[mjaa050-B5] Chen K.F. , Yen C.C., Lin J.K., et al (2015). Cancerous inhibitor of protein phosphatase 2A (CIP2A) is an independent prognostic marker in wild-type KRAS metastatic colorectal cancer after colorectal liver metastasectomy. BMC Cancer 15, 301. 25896895 10.1186/s12885-015-1300-3 PMC4404594

[mjaa050-B6] Cheng G.Z. , Zhang W., Wang L.H. (2008). Regulation of cancer cell survival, migration, and invasion by Twist: AKT2 comes to interplay. Cancer Res. 68, 957–960. 18281467 10.1158/0008-5472.CAN-07-5067

[mjaa050-B7] Clark E.A. , Golub T.R., Lander E.S., et al (2000). Genomic analysis of metastasis reveals an essential role for RhoC. Nature 406, 532–535. 10952316 10.1038/35020106

[mjaa050-B8] Ejarque M. , Altirriba J., Gomis R., et al (2013). Characterization of the transcriptional activity of the basic helix‒loop‒helix (bHLH) transcription factor Atoh8. Biochim. Biophys. Acta 1829, 1175–1183. 23938248 10.1016/j.bbagrm.2013.08.003

[mjaa050-B9] Fang F. , Wasserman S.M., Torres-Vazquez J., et al (2014). The role of Hath6, a newly identified shear-stress-responsive transcription factor, in endothelial cell differentiation and function. J. Cell Sci. 127, 1428–1440. 24463812 10.1242/jcs.136358 PMC3970556

[mjaa050-B10] Gao J. , Aksoy B.A., Dogrusoz U., et al (2013). Integrative analysis of complex cancer genomics and clinical profiles using the cBioPortal. Sci. Signal. 6, pl1. 10.1126/scisignal.2004088 PMC4160307 23550210

[mjaa050-B11] Guan X. , Chen S., Zhao Y. (2018). The role of RhoC in malignant tumor invasion, metastasis and targeted therapy. Histol. Histopathol. 33, 255–260. 28664531 10.14670/HH-11-915

[mjaa050-B12] Hakem A. , Sanchez-Sweatman O., You-Ten A., et al (2005). RhoC is dispensable for embryogenesis and tumor initiation but essential for metastasis. Genes Dev. 19, 1974–1979. 16107613 10.1101/gad.1310805 PMC1199568

[mjaa050-B13] Ikoma T. , Takahashi T., Nagano S., et al (2004). A definitive role of RhoC in metastasis of orthotopic lung cancer in mice. Clin. Cancer Res. 10, 1192–1200. 14871999 10.1158/1078-0432.ccr-03-0275

[mjaa050-B14] Inoue C. , Bae S.K., Takatsuka K., et al (2001). Math6, a bHLH gene expressed in the developing nervous system, regulates neuronal versus glial differentiation. Genes Cells 6, 977–986. 11733035 10.1046/j.1365-2443.2001.00476.x

[mjaa050-B15] Kang Y. , Massague J. (2004). Epithelial‒mesenchymal transitions: twist in development and metastasis. Cell 118, 277–279. 15294153 10.1016/j.cell.2004.07.011

[mjaa050-B16] Kress T.R. , Sabo A., Amati B. (2015). MYC: connecting selective transcriptional control to global RNA production. Nat. Rev. Cancer 15, 593–607. 26383138 10.1038/nrc3984

[mjaa050-B17] LaGory E.L. , Giaccia A.J. (2016). The ever-expanding role of HIF in tumour and stromal biology. Nat. Cell Biol. 18, 356–365. 27027486 10.1038/ncb3330 PMC4898054

[mjaa050-B18] Lang S. , Busch H., Boerries M., et al (2017). Specific role of RhoC in tumor invasion and metastasis. Oncotarget 8, 87364–87378. 29152087 10.18632/oncotarget.20957 PMC5675639

[mjaa050-B19] Lynn F.C. , Sanchez L., Gomis R., et al (2008). Identification of the bHLH factor Math6 as a novel component of the embryonic pancreas transcriptional network. PLoS One 3, e2430. 18560595 10.1371/journal.pone.0002430 PMC2413403

[mjaa050-B20] Massari M.E. , Murre C. (2000). Helix‒loop‒helix proteins: regulators of transcription in eucaryotic organisms. Mol. Cell. Biol. 20, 429–440. 10611221 10.1128/mcb.20.2.429-440.2000 PMC85097

[mjaa050-B21] Murre C. , Bain G., van Dijk M.A., et al (1994). Structure and function of helix‒loop‒helix proteins. Biochim. Biophys. Acta 1218, 129–135. 8018712 10.1016/0167-4781(94)90001-9

[mjaa050-B22] Rankin E.B. , Giaccia A.J. (2016). Hypoxic control of metastasis. Science 352, 175–180. 27124451 10.1126/science.aaf4405 PMC4898055

[mjaa050-B23] Rawnsley D.R. , Xiao J., Lee J.S., et al (2013). The transcription factor atonal homolog 8 regulates Gata4 and friend of Gata-2 during vertebrate development. J. Biol. Chem. 288, 24429–24440. 23836893 10.1074/jbc.M113.463083 PMC3750143

[mjaa050-B24] Renjie W. , Haiqian L. (2015). MiR-132, miR-15a and miR-16 synergistically inhibit pituitary tumor cell proliferation, invasion and migration by targeting Sox5. Cancer Lett. 356, 568–578. 25305447 10.1016/j.canlet.2014.10.003

[mjaa050-B25] Ridley A.J. (2006). Rho GTPases and actin dynamics in membrane protrusions and vesicle trafficking. Trends Cell Biol. 16, 522–529. 16949823 10.1016/j.tcb.2006.08.006

[mjaa050-B26] Ross M.D. , Martinka S., Mukherjee A., et al (2006). Math6 expression during kidney development and altered expression in a mouse model of glomerulosclerosis. Dev. Dyn. 235, 3102–3109. 16937370 10.1002/dvdy.20934 PMC2203212

[mjaa050-B27] Shah N. , Jin K., Cruz L.A., et al (2013). HOXB13 mediates tamoxifen resistance and invasiveness in human breast cancer by suppressing ERα and inducing IL-6 expression. Cancer Res. 73, 5449–5458. 23832664 10.1158/0008-5472.CAN-13-1178 PMC3967590

[mjaa050-B28] Siegel R.L. , Miller K.D., Jemal A. (2019). Cancer statistics, 2019. CA Cancer J. Clin. 69, 7–34. 30620402 10.3322/caac.21551

[mjaa050-B29] Skinner M.K. , Rawls A., Wilson-Rawls J., et al (2010). Basic helix‒loop‒helix transcription factor gene family phylogenetics and nomenclature. Differentiation 80, 1–8. 20219281 10.1016/j.diff.2010.02.003 PMC2894270

[mjaa050-B30] Song Y. , Pan G., Chen L., et al (2015). Loss of ATOH8 increases stem cell features of hepatocellular carcinoma cells. Gastroenterology 149, 1068–1081.e5. 26099525 10.1053/j.gastro.2015.06.010

[mjaa050-B31] Stine Z.E. , Walton Z.E., Altman B.J., et al (2015). MYC, metabolism, and cancer. Cancer Discov. 5, 1024–1039. 26382145 10.1158/2159-8290.CD-15-0507 PMC4592441

[mjaa050-B32] Vainio P. , Lehtinen L., Mirtti T., et al (2011). Phospholipase PLA2G7, associated with aggressive prostate cancer, promotes prostate cancer cell migration and invasion and is inhibited by statins. Oncotarget 2, 1176–1190. 22202492 10.18632/oncotarget.397 PMC3282076

[mjaa050-B33] Wan L. , Pantel K., Kang Y. (2013). Tumor metastasis: moving new biological insights into the clinic. Nat. Med. 19, 1450–1464. 24202397 10.1038/nm.3391

[mjaa050-B34] Wang D. , Han S., Wang X., et al (2015). SOX5 promotes epithelial‒mesenchymal transition and cell invasion via regulation of Twist1 in hepatocellular carcinoma. Med. Oncol. 32, 461. 25572815 10.1007/s12032-014-0461-2

[mjaa050-B35] Wang Z. , Xie J., Yan M., et al (2016). Downregulation of ATOH8 induced by EBV-encoded LMP1 contributes to the malignant phenotype of nasopharyngeal carcinoma. Oncotarget 7, 26765–26779. 27049918 10.18632/oncotarget.8503 PMC5042013

[mjaa050-B36] Yamamoto N. , Nishikawa R., Chiyomaru T., et al (2015). The tumor-suppressive microRNA-1/133a cluster targets PDE7A and inhibits cancer cell migration and invasion in endometrial cancer. Int. J. Oncol. 47, 325–334. 25955017 10.3892/ijo.2015.2986

