# Supplementary material for: A novel isoform of ATOH8 promotes the metastasis of breast cancer by regulating RhoC
Source: J Mol Cell Biol. 2020 Oct 13;13(1):59–71. doi: 10.1093/jmcb/mjaa050 (PMC8035989; doi:10.1093/jmcb/mjaa050)
Supplement: mjaa050_Supplementary_Data [file mjaa050_supplementary_data.pdf]

Supplementary material

A novel isoform of ATOH8 promotes the metastasis of breast cancer by regulating RhoC

Running title: ATOH8 isoform promotes breast cancer metastasis

Mengyao Xu[1,†], Shan Huang[1,3,†], Xiaoli Dong[1,†], Yanan Chen[1,2], Miao Li[1], Wen Shi[1], Guanwen Wang[1], Chongbiao Huang[4], Qiong Wang[1], Yanhua Liu[1,2], Peiqing Sun[3], Shuang Yang[1], Rong Xiang[1,2], and Antao Chang[1,2,3,*]

[1] School of Medicine, Nankai University, Tianjin 300071, China

[2] International Collaborative Innovation Center of Medicine, Nankai University, Tianjin 300071, China

[3] Department of Cancer Biology and Comprehensive Cancer Center, Wake Forest University Medical Center, Winston-Salem, NC 27157, USA

[4] Department of Pancreatic Cancer, Tianjin Medical University Cancer Institute and Hospital, National Clinical Research Center for Cancer, Key Laboratory of Cancer Prevention and Therapy, Tianjin 300060, China

[†] These authors contributed equally to this work.

* Correspondence to: Antao Chang, E-mail: changantao@nankai.edu.cn, anchang@wakehealth.edu; Tel: +86-022-2350-9557

Supplementary Materials

Antibodies

Antibody against ATOH8 (Cat. # AV39728) was purchased from Sigma-Aldrich. Antibody against RhoC (Cat. # ab180785) for IF (Immunofluorescence) and IHC (Immunohistochemistry) was purchased from Abcam. Antibody against RhoC (Cat. # 3430), N-Cadherin (Cat. # 13116S) and Vimentin (Cat. # 5741S) for Western Blot were purchased from Cell Signaling Technology. Antibody against Twist (Cat. # ab175430) and Zeb1 (Cat. # ab203829) for Western Blot were purchased from Abcam. Antibody against E-Cadherin (Cat. # 610181) was purchased from BD. Antibody against β-actin (Cat. # sc-47778) for Western Blot was purchased from Santa Cruz Biotechnology.

Antibody specifically against ATOH8-V1 was produced by GenScript using a synthetic 14aa peptide (NANRHCSRPLPPPS) from its unique C-terminal domain as antigen for immunity to rabbit.

Lentivirus production

Stocks of lentiviruses encoding cDNA or shRNA were produced by transfecting a four plasmids system into HEK-293T cells, which included the pLV-cDNA/shRNA vector and the helper vectors (Gag-Pol, VSV-G, and REV). The procedure of lentivirus production was summarized as following:

Day 0: Seed 1×10^6 293T cells in a 6-well plate, so that the cell density will be around 80-90% at the time of transduction.

Day 1: Gently mix 7.5μl Lipofectamine™ 2000 (Invitrogen) and 0.25ml Opti-MEM medium (Gibco) and incubate at room temperature for 5 minutes. Meanwhile, gently mix 1.5μg pLV-cDNA/shRNA vector and 1.5μg packaging vectors mixture (0.75μg Gag-Pol, 0.3μg Rev and 0.45μg VSV-G) with 0.25ml Opti-MEM medium. Gently mix the two components and incubate for 20 minutes, and then add into the cells containing 1 ml fresh complete medium.

Day 2: Carefully replace the medium with 3ml complete medium at 12-16 hours post-transfection.

Day 3: Collect supernatants into a polypropylene storage tube at 40-48 hours post-transfection and store at -80 ℃.

Lentivirus infection

Day 0: Seed 1×10^5 breast cancer cells in a 6-well plate, so that the cell density will be around 15-25% at the time of transduction.

Day 1: Replace the medium with 1-2ml fresh complete medium (without Penicillin/ Streptomycin) containing 8μg/ml polybrene (final concentration). Gently mix the lentivirus stock and add 1ml

virus to each well. Spin transduction in a desktop centrifuge at 1,600 rpm for 60min at room temperature. Replace the transduction medium with 2ml fresh complete medium.

Day 3: Select transduced cells with medium containing appropriate antibiotics for stable cell lines (5-10μg/ml Blasticidin for gene expression, 2-5μg/ml Puromycin for shRNA).

Day 6+: Expand the culture of cell lines stably expressing target gene or shRNA, and store the cell line stocks in liquid nitrogen. Analysis of transduced cells by qPCR and western blotting.

Western blotting

Protein samples from breast cancer cell lines were isolated by RIPA lysis buffer containing 1×Protease Inhibitor cocktail, 1×Phosphatase Inhibitor Cocktail 2 and 1×Phosphatase Inhibitor Cocktail 3 (Sigma-Aldrich), and quantitated by BCA Protein Assay Kit (ThermoFisher Scientific). Then, protein lysates were diluted to 2.5μg/μl with loading buffer and boiled for 10 minutes. 15 to 40μg of proteins were resolved on 10% or 12% SDS-polyacrylamide gels and then transferred to the PVDF membrane (Millipore) using the Bio-Rad Electrophoresis system. After blocking in TBST containing 5% fetal bovine serum (FBS) for 1h, the filters were incubated with primary antibodies (1:1000 in TBST containing 5% FBS) followed by horseradish peroxidase (HRP)-conjugated secondary antibodies (Santa Cruz Biotechnology, 1:5000 in TBST containing 5% skim milk), and visualized by ECL (enhanced chemiluminescence, Millipore). Signals were captured by G: BOX Chemi XRQ gel doc system (Syngene) or photographic film.

Immunofluorescence analysis

1×10^5 breast cancer cells were seeded in a sterilized glass bottom microwell dish (MatTek Corporation) and cultured for two days, so that the cell density will be around 60–80% at the time of staining. Cells were fixed in 4% paraformaldehyde and permeated by 0.05% Triton X-100 for 10 minutes. After gently washed with PBS for 3 times, cells were blocked in PBS containing 5% goat serum for 1h, and then incubated with an antibody that recognizes ATOH8 (1:50 in PBS containing 5% goat serum), or ATOH8-V1 (1:100), or RhoC (1:100) at 4 ℃ for overnight. At the next day, cells were washed 3 times with PBS, each for 10 min, and incubated with a fluorescence-labeled secondary antibody (1:200 in PBS containing 5% goat serum) for 1 hour at room temperature in the dark. Then cell nucleus were stained with DAPI (1:1000 in PBS) for 2 minutes at room temperature. After washing in PBS, cells were visualized by the laser scanning confocal microscope (Olympus, FV1000).

The fluorescence-labeled secondary antibodies used in the study are as following: Alexa Fluor 488-Donkey anti-Rabbit IgG for ATOH8, and Alexa Fluor 594-Donkey anti-Rabbit IgG for ATOH8-V1 and RhoC were purchased from Life Technologies.

Cell proliferation assay

Breast cancer cells with indicated genotypes were seeded into a 96-well plate at a density of 5×10^3 cells/well, and cultured for indicated time. Cell viability was then assessed using the CCK-8 assay (Dojindo Molecular Technologies) according to the manufacturer's protocols.

Apoptosis analysis by flow cytometry

Breast cancer cells were transfected with pLV-ATOH8-V1 or pLV-ATOH8 expression vectors, or empty vector as control using Lipofectamine™ 2000 and cultured for 48 hours. Collected the cells and washed them twice with cold PBS, and then resuspend cells in 1×Binding Buffer (BD Biosciences) at a concentration of 1×10^6 cells/ml. Transferred 100μl cell suspension into a 5ml culture tube and incubated with 5μl FITC-Annexin V and 10μl PI (BD Biosciences) for 15 minutes in the dark. Add 400μl of 1×Binding Buffer to each tube, and then analyze by flow cytometry immediately.

Supplementary Tables

Supplementary Table 1 Sequences of the primers used for qPCR and ChIP assay.

**Gene**		**Sequence (5' to 3')**	**Application**
ATOH8	F	GCCTCCTACGAGATCAAAGCC	qPCR
	R	TGGTCCGCACTGTAGTCAAGGT	
ATOH8-V1	F	CAATAAGGGCAAGCACTTCAGC	qPCR
	R	ATGTGCTTAGTGGTGGCGTGG	
RhoC	F	GACACAGCAGGGCAGGAAGACT	qPCR
	R	GCACGGGCTCCTGCTTCATC	
Sox5	F	ATGCCTGTTATCCAGAGCACTTAC	qPCR
	R	TGGCTTGTCCTGCAATATGGT	
PLA2G7	F	TATACGAAATGAGCAGGTACGGC	qPCR
	R	CTTCATCACCCAGTGGAAACATC	
PDE7A	F	TTTAGGTCCCATTTGGATAGAGG	qPCR
	R	TGGATGTTGGCAATAGATTCAGT	
HoxB13	F	AGTGAGAACAGAACCCACCAGG	qPCR
	R	GATGAACTTGTTAGCCGCATACTC	
GAPDH	F	GTGGCAAAGTGGAGATTGTT	qPCR
	R	CTCGCTCCTGGAAGATGG	
RhoC	F	CTGGAGCCTGCTTGTGTTGAG	ChIP
	R	GAATGAATCCGTTCTGGAGATACC	

F, Forward primer; R, Reverse primer.

Supplementary Table 2 Sequences of primers used for vector construction.

**Gene**		**Sequence (5' to 3')**	**Enzyme**
ATOH8	F	CGGGATCC*GCCACC*ATGAAGCACATCCCGGTCCT	BamHI
	R	CGACGCGTTCACTCCTTGCGCTTCTTGG	MluI
ATOH8-V1	F	CGGGATCC*GCCACC* ATGAAGCACATCCCGGTCCTC	BamHI
	R	CGACGCGTTCACCACATGGTTGATGTGCTTG	MluI
RhoC	F	GCTCTAGA*GCCACC*ATGGCTGCAATCCGAAAGAAG	XbaI
	R	CGGGATCCGTTGTAGGGGGATAATTTCTGTACC	BamHI
RhoC-promoter	F	GAAGATCTCTGAATGTAGTGGTGAGTGCCTGTG	BglII
	R	CCCAAGCTTAGCAGGGTAATTCAGACGGCAC	HindIII
RhoC-promoter-dt	F	CACCCTACACCTAGGGAAGGAATTATTCCCGCCATACCTCCCTC	
	R	GAGGGAGGTATGGCGGGAATAATTCCTTCCCTAGGTGTAGGGTG	

F, Forward primer; R, Reverse primer.

For expression, amplified fragments of gene coding region were inserted into the pLV-cDNA cloning vector (Biosettia). For dual luciferase assay, amplified fragments of gene promoter were inserted into the pGL3-Basic vector (Promega).

The deletion of ATOH8-V1 binding region on RhoC promoter was generated by a 2-step PCR method. The first-step PCR was performed with two pair of primers (RhoC-promoter F/RhoC-promoter-dt R; RhoC-promoter-dt F/RhoC-promoter R) to amplify up-stream and down-stream fragments of the deletion region. These two fragments have a 44bp overlap (3' end of the up-stream and 5' end of the down-stream fragments), so a 1:1 mixture of them can be served as the template for the second-step PCR with primers RhoC-promoter F/ R to generate the target fragment for cloning.

Supplementary Table 3 shRNA sequences for gene silencing.

Gene	shRNA	Sequence (5' to 3')
ATOH8-V1	sh1	*AAAA*GTCCTGCACCCTTAGCATA*TTGGATCCAA*TATGCTAAGGGTGCAGGAC
	sh2	*AAAA*GCCTGCAGCCCATCCAGAAAT*TTGGATCCAA*ATTTCTGGATGGGCTGCAGGC
	sh3	*AAAA*GGGAGTCATACCAAGAAACAT*TTGGATCCAA*ATGTTTCTTGGTATGACTCCC
	sh4	*AAAA*GCATAACATGTGGTCATAT*TTGGATCCAA*ATATGACCACATGTTATGC
	sh5	*AAAA*CACCAAGCACATCAACCAT*TTGGATCCAA*ATGGTTGATGTGCTTGGTG
RhoC	sh1	*AAAA*ATAAGAAGGACCTGAGGCA*TTGGATCCAA*TGCCTCAGGTCCTTCTTAT
	sh2	*AAAA*CATGTGCTTCTCCATCGAC*TTGGATCCAA*GTCGATGGAGAAGCACATG
Control	SC	*AAAA*GCAGTTATCTGGAAGATCAGG*TTGGATCCAA*CCTGATCTTCCAGATAACTGC

shRNA is designed using the online tool of Biossetia Company. SC (shRNA control) targetes the β-galactosidase gene from *E.coli*. To silence ATOH8-V1, 3 shRNAs with different combinations were co-infected into the cells to achieve high efficiency.

Supplementary Figures

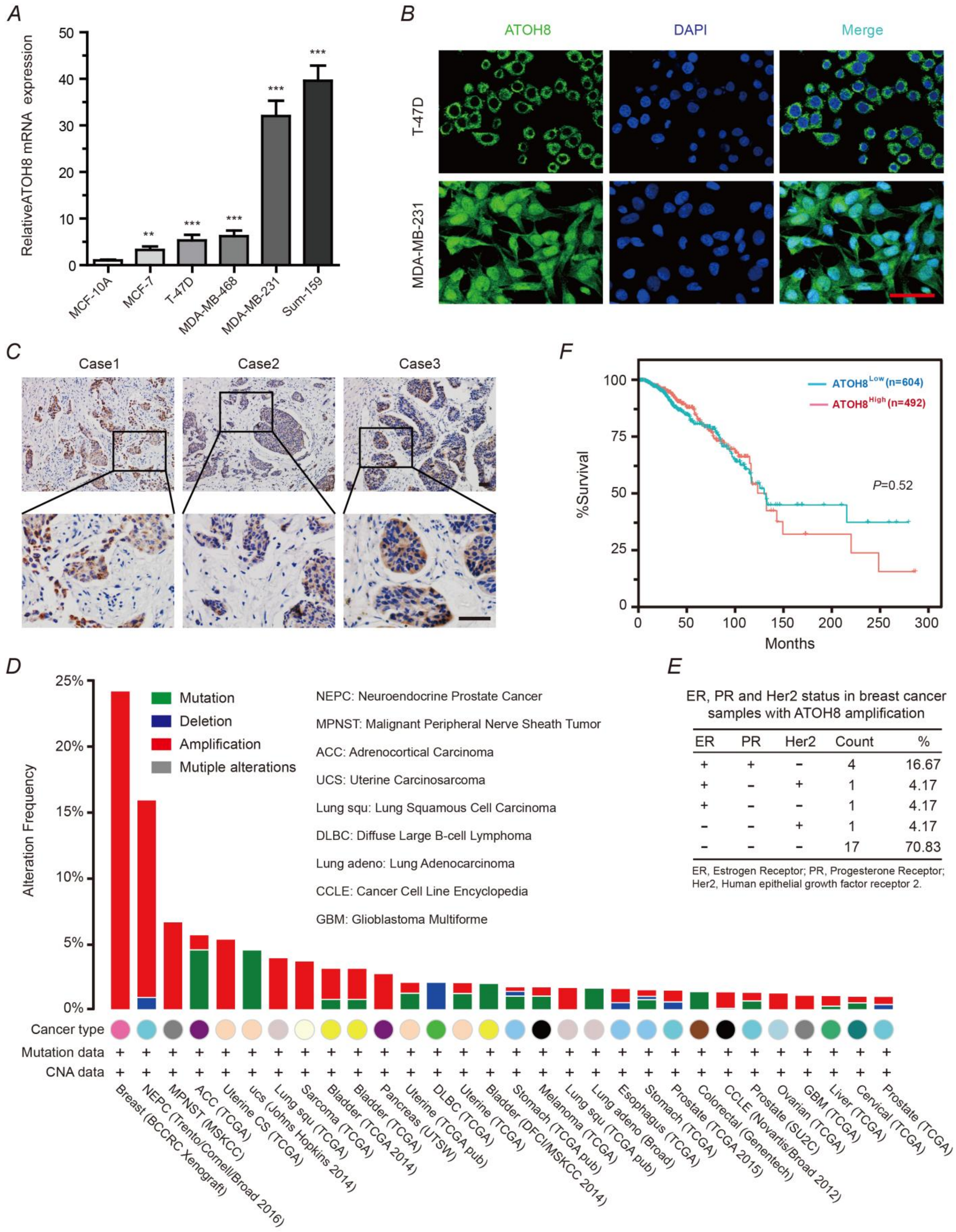

Supplementary Figure 1 ATOH8 is highly expressed in breast cancer.

A, Real-time qPCR analysis of ATOH8 mRNA levels in breast cancer cell lines. ***p*<0.01 and ****p*<0.001 for comparisons to the first lane using unpaired *t*-test.

B, Immunofluorescent staining of ATOH8 in T-47D and MDA-MB-231 cells.

C, Immunohistochemical staining of ATOH8 in breast cancer tissues.

D, Cross-cancer alteration summary for ATOH8 using the cBioPortal.

E, Analysis of ER, PR and Her2 status in breast cancer samples with ATOH8 amplification from the cBioPortal.

F, Survival analysis of breast cancer patients with ATOH8 high expression or low expression from TCGA database.

Kaplan-Meier analysis is used for the estimation of overall survival. *p* value was assessed by the log-rank test. All scale bars represent 100 μm.

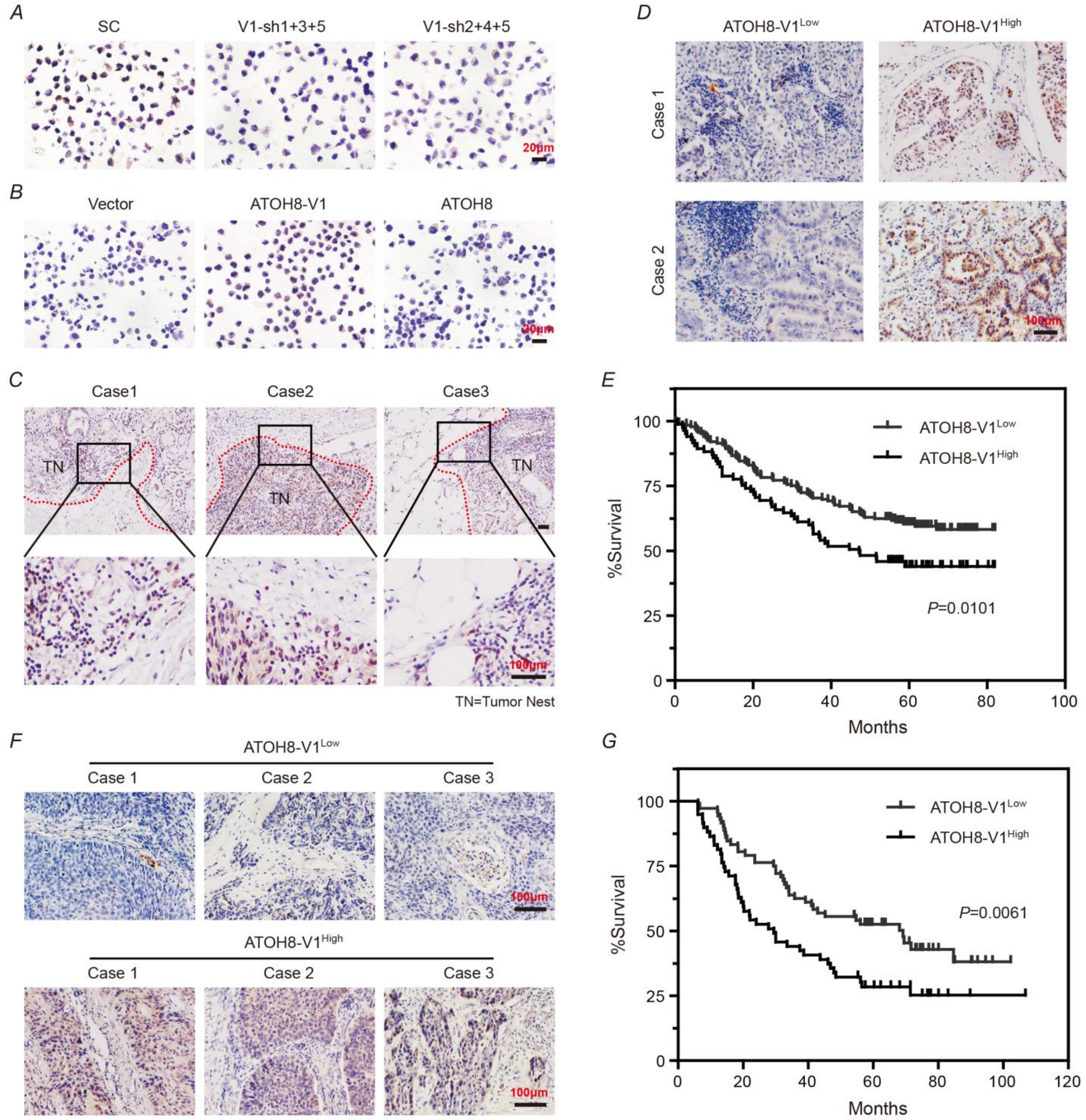

Supplementary Figure 2 ATOH8-V1 is highly expressed in lung cancer and esophageal cancer, and is a negative prognostic indicator of overall survival of patients.

A-*B*, Verification of ATOH8-V1 antibody used for immunohistochemical staining in MDA-MB-231 (*A*) and T-47D (*B*) cell culture blocks.

C, Immunohistochemical staining of ATOH8-V1 in lung cancer tissues.

D, The criterion for high and low ATOH8-V1 expression in lung cancer tissues.

E, Survival analysis of lung cancer patients with high and low ATOH8-V1 expression.

F, The criterion for high and low ATOH8-V1 expression in esophageal cancer tissues.

G, Survival analysis of esophageal cancer patients.

Kaplan-Meier analysis is used for the estimation of overall survival. *p* value was assessed by the log-rank test.

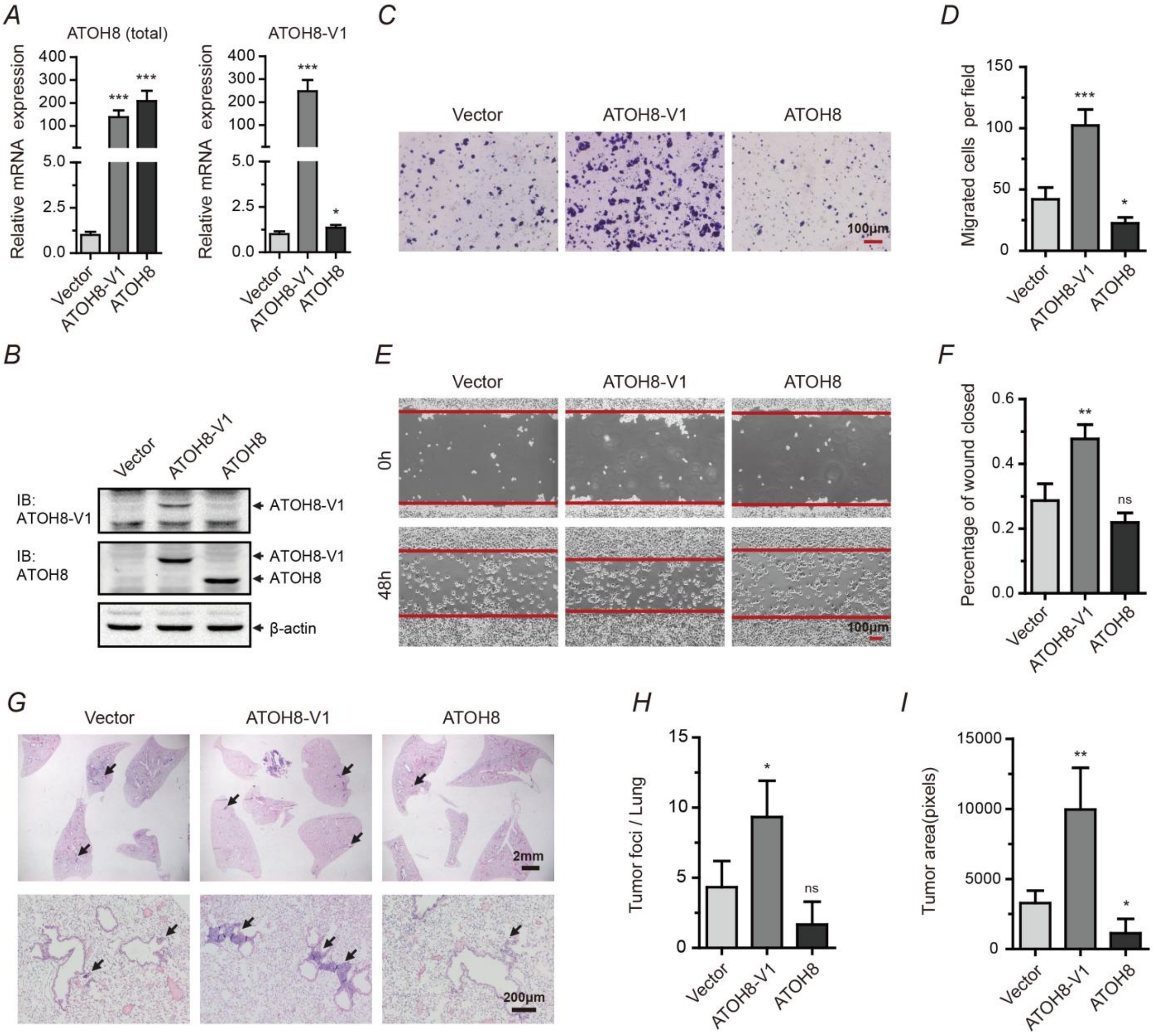

Supplementary Figure 3 Forced expression of ATOH8-V1 promotes metastasis of MDA-MB-468 cells.

A-*B*, qPCR (*A*) and immunoblotting analysis (*B*) of ATOH8-V1 and ATOH8 in MDA-MB-468 cells transduced with ATOH8-V1 or ATOH8.

C-*D*, Trans-well assay detecting invasion of MDA-MB-468 cells with ATOH8-V1 or ATOH8 over expression.

E-*F*, Wound healing assay detecting migration of MDA-MB-468 with ATOH8-V1 or ATOH8 over expression.

G, 5×10^6 MDA-MB-468 cells transduced with ATOH8-V1 or ATOH8 were subcutaneously injected in the second fat pad of female NOD-SCID mice. Orthotopic xenograft tumors were surgically removed when they were 500-600 mm^3 in size. 12 weeks after injection, mice were scarified and lung tissues were collected for H&E staining to detect lung metastasis, n=6.

H, Quantification of tumor foci numbers in lung tissues of mice.

I, Quantification of tumor area in lung tissues of mice.

ns, not significant, $p>0.05$, $*p<0.05$, $**p<0.01$, and $***p<0.001$ for comparisons with Vector groups using unpaired *t*-test.

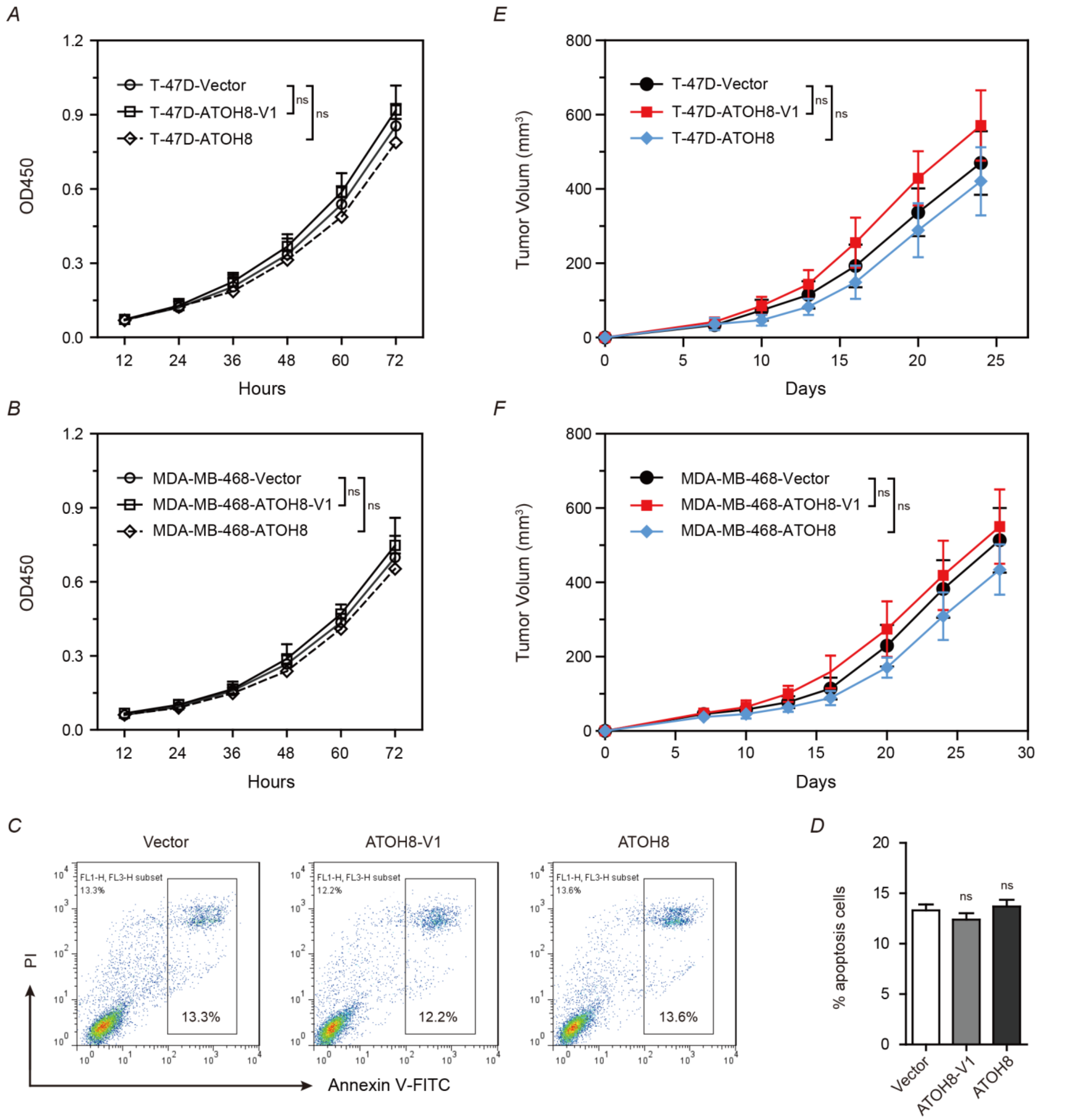

Supplementary Figure 4 ATOH8-V1 has no effect on proliferation and apoptosis of breast cancer cell lines.

A-B, Cell proliferation analysis of T-47D (*A*) and MDA-MB-468 (*B*) cells transduced with ATOH8-V1 or ATOH8 using CCK8. ns, not significant, p>0.05 in unpaired *t*-test.

C-D, Apoptosis analysis of T-47D cells with ATOH8-V1 or ATOH8 over expression by FACS. ns, not significant, p>0.05 for comparisons with Vector groups using unpaired *t*-test.

E-F, Tumor growth curve of T-47D (*E*) and MDA-MB-468 (*F*) cells transduced with ATOH8-V1 or ATOH8 in NOD-SCID mice. 5×10^6 T-47D or MDA-MB-468 cells transduced with ATOH8-V1 or ATOH8 were subcutaneously injected in the second fat pad of female NOD-SCID mice, n=6. Tumor volumes were measured every 3-4 days. Tumor volume was calculated by $\text{Length}\times\text{Width}^2/2$. ns, not significant, $p>0.05$ in unpaired *t*-test.

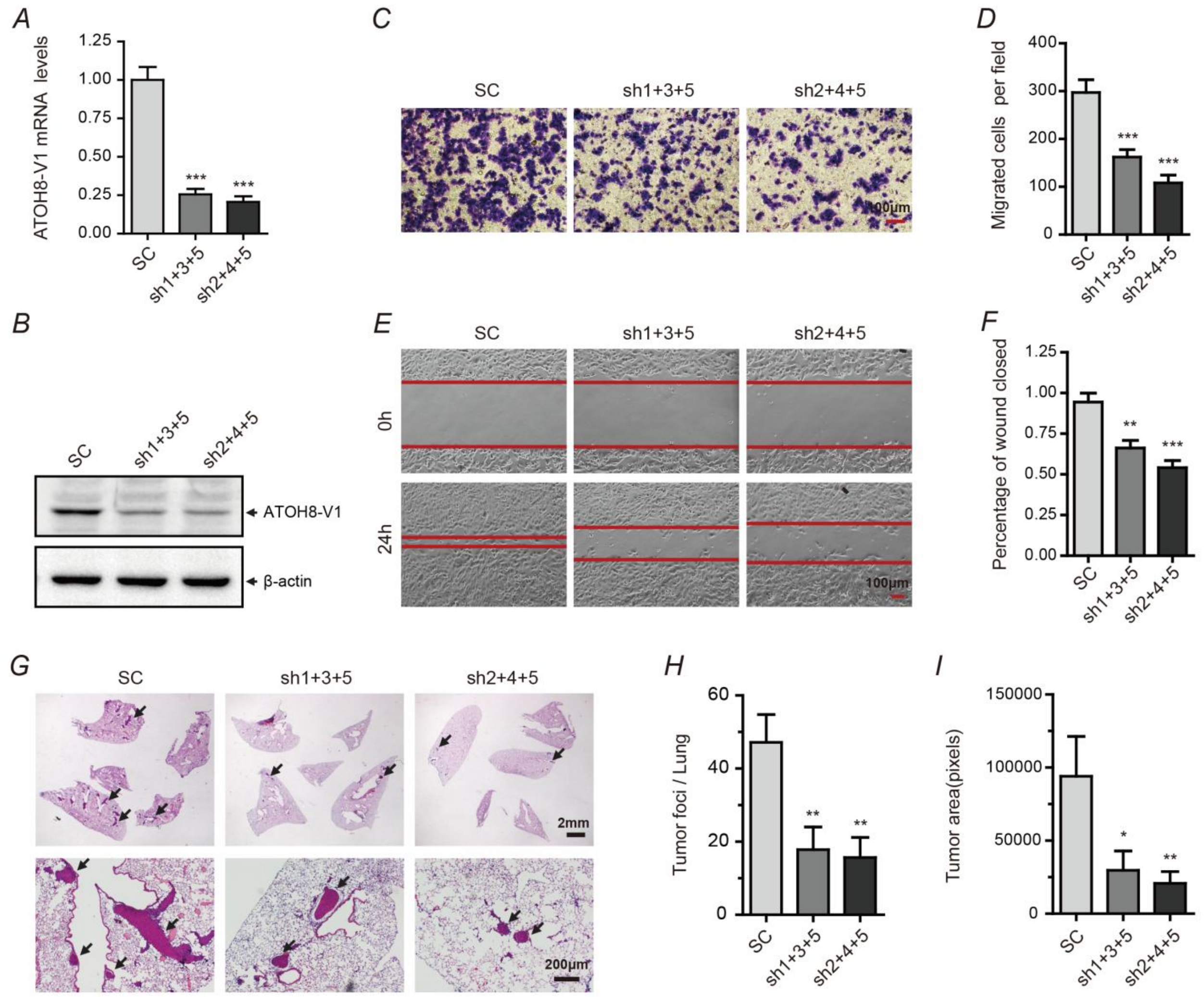

Supplementary Figure 5 Silencing of ATOH8-V1 inhibits metastasis of Sum-159 cells.

A-*B*, qPCR (*A*) and immunoblotting analysis (*B*) of the knock-down efficiency of ATOH8-V1 in Sum-159 cells transduced with ATOH8-V1 shRNAs.

C-*D*, Trans-well assay detecting invasion of Sum-159 cells with ATOH8-V1 knock down.

E-*F*, Wound healing assay detecting migration of Sum-159 cells with ATOH8-V1 knock down.

G, 1×10^6 Sum-159 cells transduced with ATOH8-V1 shRNAs were subcutaneously injected in the second fat pad of female NOD-SCID mice. Orthotopic xenograft tumors were surgically removed when they were 500-600 mm^3 in size. 9 weeks after injection, mice were scarified and lung tissues were collected for H&E staining to detect lung metastasis, n=6.

H, Quantification of tumor foci numbers in lung tissues of mice.

I, Quantification of tumor area in lung tissues of mice.

*p<0.05, **p<0.01, and ***p<0.001 for comparisons to SC groups in unpaired t-test.

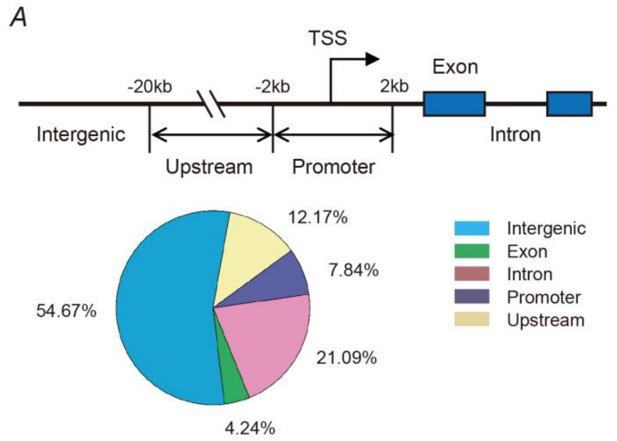

B

5 candidate downstream effectors of ATOH8-V1 that are associated with tumor metastasis

Symbol	Chr.	Strand	Length	-10 Log (p)	Fold-enrichment
SoX5	12	-	352	1953.99	57.14
RhoC	1	-	400	2562.13	23.5
PLA2G7	6	-	346	2156.38	22.7
PDE7A	8	-	324	453.97	17.5
HoxB13	17	-	327	1292.44	13.92

Chr., Chromone; -10 Log (p), -10 Log (p-value).

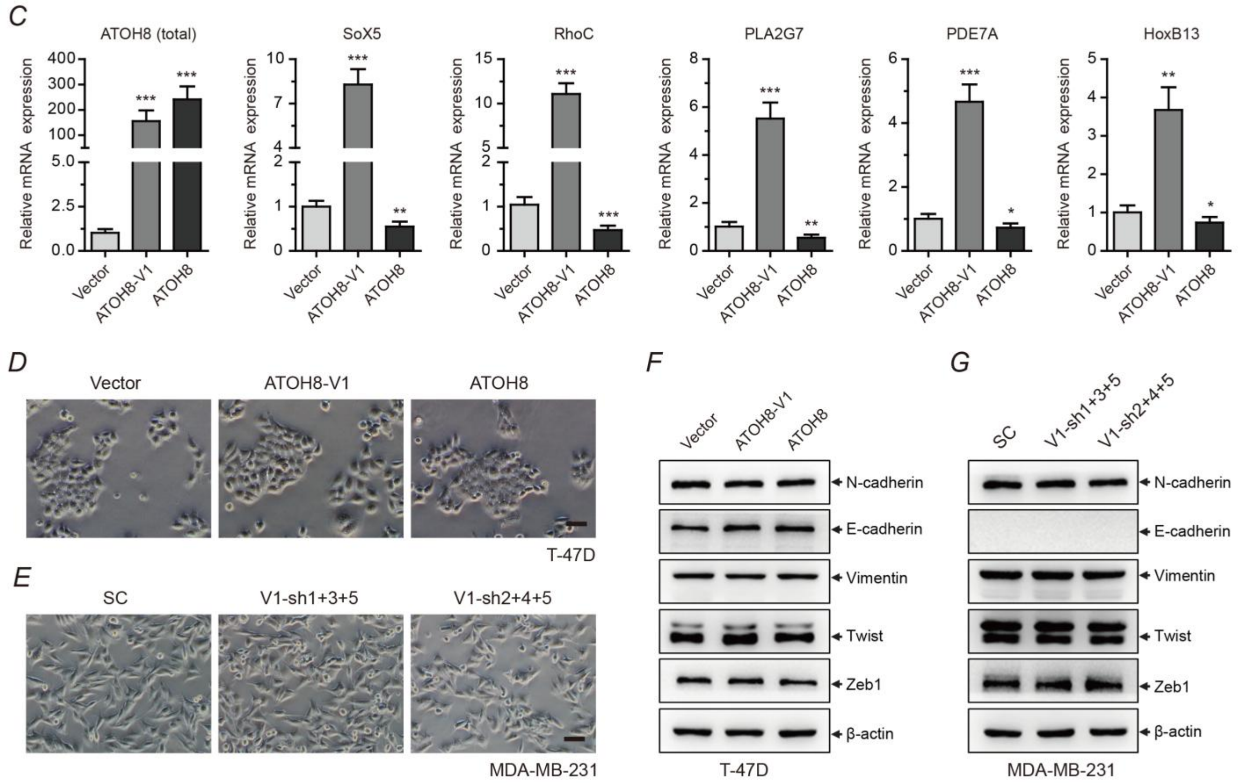

Supplementary Figure 6 ChIP-Seq analysis of downstream effectors of ATOH8-V1 in breast cancer cell lines.

A, Analysis of the location of ATOH8-V1 binding sites (identified by ChIP-Seq analysis using the anti-ATOH8-V1 antibody) in the genome.

B, Potential ATOH8-V1 downstream effectors that are associated with tumor metastasis in ChIP-Seq analysis.

C, qPCR analysis of the mRNA levels of the metastasis-related ATOH8-V1 downstream effectors in T-47D cells with ATOH8-V1 or ATOH8 over expression. *p<0.05, **p<0.01, and ***p<0.001 for comparisons with the first lane in unpaired t-test.

D, Cell morphology of T-47D cells transduced with ATOH8-V1 or ATOH8.

E, Cell morphology of MDA-MB-231 cells transduced with ATOH8-V1 shRNA.

F, Immunoblotting analysis of EMT markers in T-47D cells transduced with ATOH8-V1 or ATOH8.

G, Immunoblotting analysis of EMT markers in MDA-MB-231 cells transduced with ATOH8-V1 shRNAs.

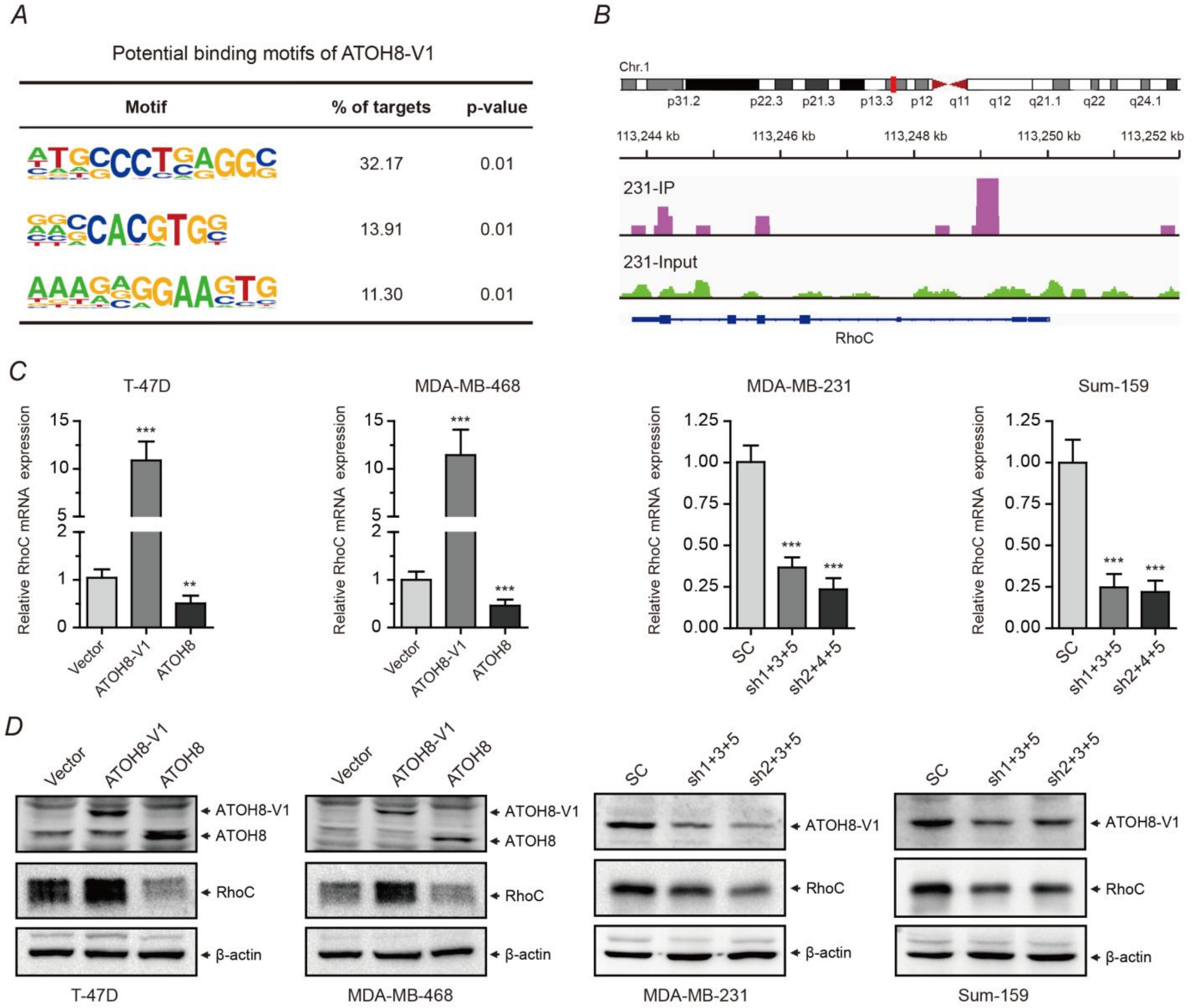

Supplementary Figure 7 ATOH8-V1 directly regulates the expression of RhoC in breast cancer cell lines.

A, Potential binding motifs of ATOH8-V1 identified by ChIP-Seq analysis using the anti-ATOH8-V1 antibody.

B, The binding region of ATOH8-V1 in the RhoC promoter from ChIP-Seq analysis.

C, qPCR analysis of RhoC mRNA levels in breast cancer cell lines with ATOH8-V1 or ATOH8 over expression, or ATOH8-V1 knockdown. **p<0.01 and ***p<0.001 for comparisons to the first lane of each group using unpaired t-test.

D, Immunoblotting analysis of ATOH8-V1 and RhoC in breast cancer cell lines with ATOH8-V1 or ATOH8 over expression, or ATOH8-V1 knockdown.

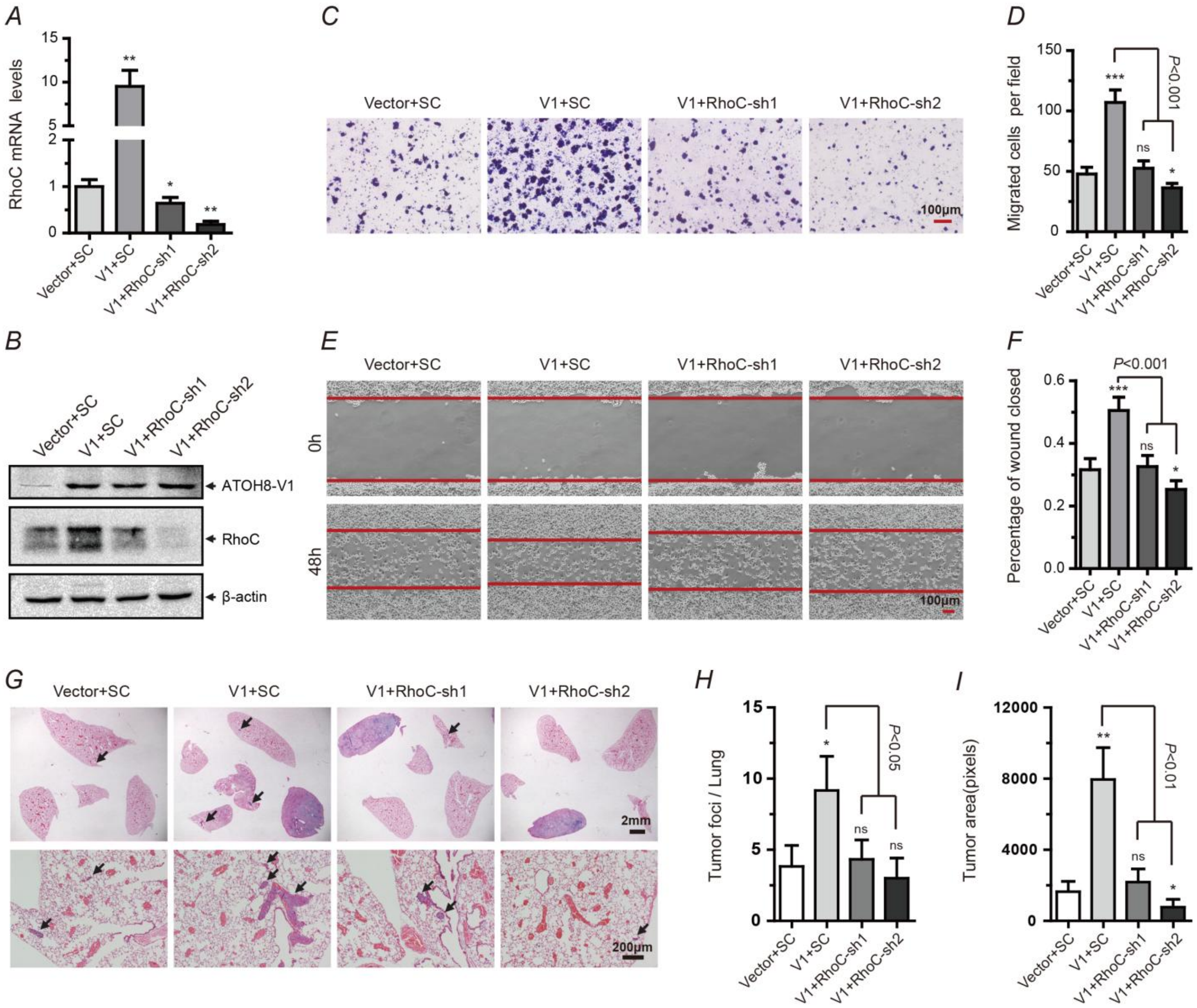

Supplementary Figure 8 Silencing of RhoC blocks the promotion effect of ATOH8-V1 on metastasis of MDA-MB-468 cells.

A, qPCR analysis of RhoC mRNA levels in ATOH8-V1 over expressed MDA-MB-468 cells transduced with RhoC shRNA.

B, Immunoblotting analysis of ATOH8-V1 and RhoC in ATOH8-V1 over expressed MDA-MB-468 cells transduced with RhoC shRNA.

C-D, Trans-well assay detecting invasion of ATOH8-V1 over expressed MDA-MB-468 cells transduced with RhoC shRNA.

E-F, Wound healing assay detecting migration of ATOH8-V1 over expressed MDA-MB-468 cells transduced with RhoC shRNA.

G, H&E staining of lung tissue slices detecting lung metastasis of ATOH8-V1 over expressed MDA-MB-468 cells transduced with RhoC shRNA in mice, n=6.

H, Quantification of tumor foci numbers in lung tissues of mice.

I, Quantification of tumor area in lung tissues.

ns, not significant, p>0.05, *p<0.05, **p<0.01, and ***p<0.001 for comparisons with Vector+SC groups using unpaired t-test.

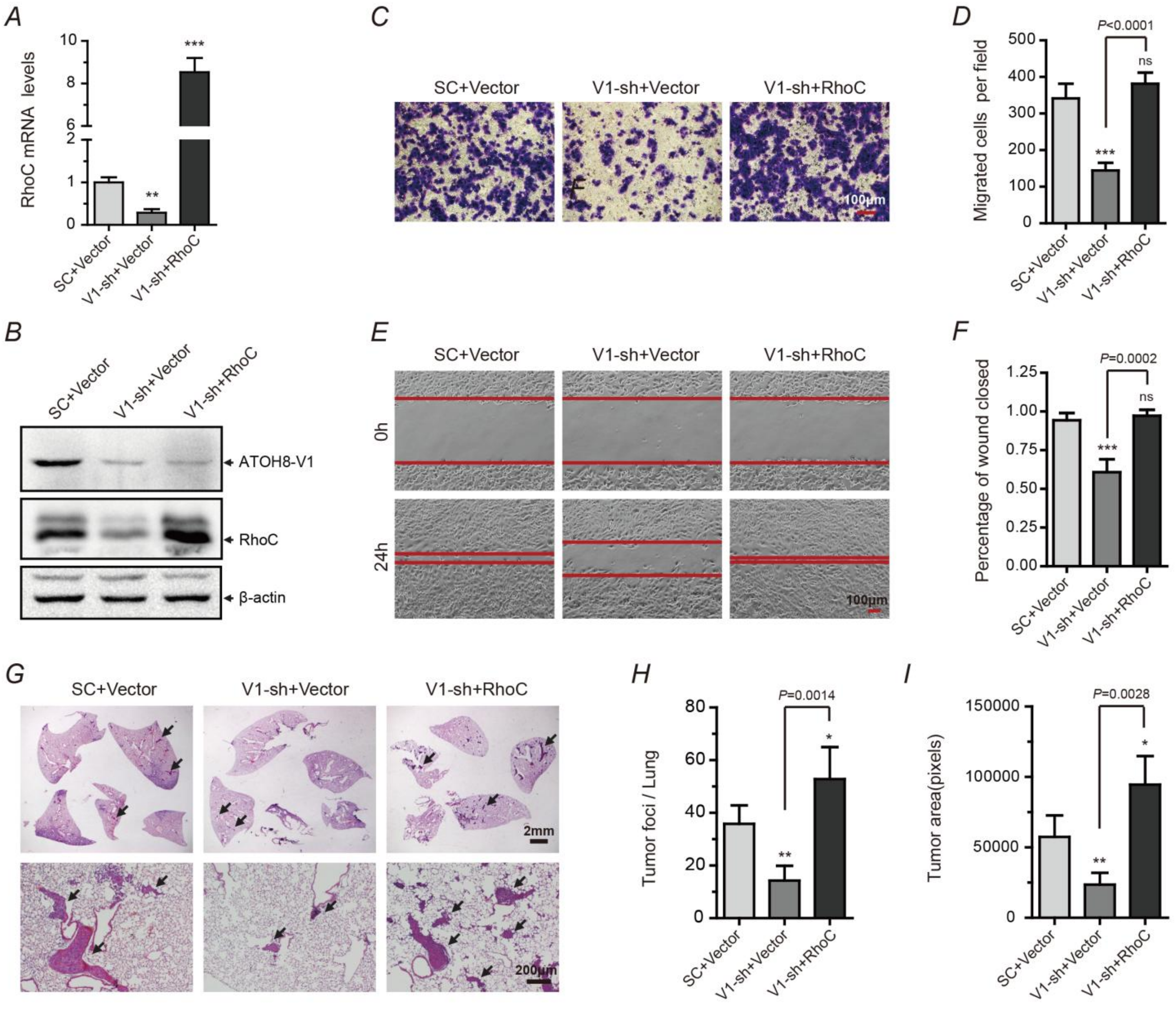

Supplementary Figure 9 Forced expression of RhoC recovers the metastatic ability of ATOH8-V1 silenced Sum-159 cells.

A, qPCR analysis of RhoC in ATOH8-V1 silenced Sum-159 cells transduced with RhoC.

B, Immunoblotting analysis of ATOH8-V1 and RhoC in ATOH8-V1 silenced Sum-159 cells transduced with RhoC.

C-D, Trans-well assay detecting invasion of ATOH8-V1 silenced Sum-159 cells transduced with RhoC.

E-F, Wound healing assay detecting migration of ATOH8-V1 silenced Sum-159 cells transduced with RhoC.

G, H&E staining of lung tissue slices detecting lung metastasis of ATOH8-V1 silenced Sum-159 cells transduced with RhoC in mice, n=6.

H, Quantification of tumor foci numbers in lung tissues of mice.

I, Quantification of tumor area in lung tissues of mice.

ns, not significant, $p>0.05$, $^*p<0.05$, $^{**}p<0.01$, and $^{***}p<0.001$ for comparisons with SC+ Vector groups in unpaired *t*-test.
